# Interaction of PINK1 with nucleotides and kinetin

**DOI:** 10.1126/sciadv.adj7408

**Published:** 2024-01-19

**Authors:** Zhong Yan Gan, Sylvie Callegari, Thanh N. Nguyen, Nicholas S. Kirk, Andrew Leis, Michael Lazarou, Grant Dewson, David Komander

**Affiliations:** ^1^ Walter and Eliza Hall Institute of Medical Research, Parkville, Victoria, Australia.; ^2^ Department of Medical Biology, University of Melbourne, Melbourne, Victoria, Australia.; ^3^ Department of Biochemistry and Molecular Biology, Biomedicine Discovery Institute, Monash University, Melbourne, Australia.

## Abstract

The ubiquitin kinase PINK1 accumulates on damaged mitochondria to trigger mitophagy, and PINK1 loss-of-function mutations cause early onset Parkinson’s disease. Nucleotide analogs such as kinetin triphosphate (KTP) were reported to enhance PINK1 activity and may represent a therapeutic strategy for the treatment of Parkinson’s disease. Here, we investigate the interaction of PINK1 with nucleotides, including KTP. We establish a cryo-EM platform exploiting the dodecamer assembly of *Pediculus humanus corporis* (*Ph*) PINK1 and determine PINK1 structures bound to AMP-PNP and ADP, revealing conformational changes in the kinase N-lobe that help establish PINK1’s ubiquitin binding site. Notably, we find that KTP is unable to bind *Ph*PINK1 or human (*Hs*) PINK1 due to a steric clash with the kinase “gatekeeper” methionine residue, and mutation to Ala or Gly is required for PINK1 to bind and use KTP as a phosphate donor in ubiquitin phosphorylation and mitophagy. *Hs*PINK1 M318G can be used to conditionally uncouple PINK1 stabilization and activity on mitochondria.

## Teaser

PINK1-nucleotide complexes reveal kinase plasticity and explain why a gatekeeper mutation is required for PINK1 to use KTP.

## INTRODUCTION

Parkinson’s disease (PD) is an incurable neurodegenerative disease, affecting more than 10 million individuals globally. While exact molecular mechanisms that cause the pathophysiology of PD remain unclear, much evidence suggests that a decline in mitochondrial health is a major contributor (*1*, *2*). The ubiquitin kinase PINK1 (encoded by *PARK6*/*PINK1*) and the E3 ubiquitin ligase Parkin (encoded by *PARK2*/*PRKN*) are among >15 *PARK*-encoded proteins that, when mutated, cause an early onset form of PD (EOPD), accounting for ~5 to 10% of PD cases (*3*–*5*). PINK1 and Parkin are crucial mediators of mitophagy, a mitochondrial quality control pathway that degrades damaged mitochondria (*1*, *6*–*8*). PINK1 serves as a key damage sensor and initiator of mitophagy. The kinase rapidly turns over under basal conditions (*8*) but, upon mitochondrial depolarization, accumulates on the outer mitochondrial membrane (OMM) where it forms a complex with the translocase of the outer membrane (TOM) and activates by autophosphorylation (*9*–*13*). Active PINK1 phosphorylates ubiquitin (*14*–*18*), enabling Parkin to be recruited to mitochondria by binding to phosphorylated ubiquitin (phospho-ubiquitin). PINK1 then contributes to the activation of Parkin by phosphorylating Parkin’s ubiquitin-like (Ubl) domain, leading to Parkin-mediated ubiquitination of OMM proteins and mitophagy (*1*, *6*, *19*).

Recent structural analysis of PINK1 from the body louse *Pediculus humanus corporis* (*Ph*) and the flour beetle *Tribolium castaneum* (*Tc*) provided detailed insights into the mechanism of PINK1 activation (*12*, *13*, *20*–*23*). PINK1 harbors a bilobal kinase fold, comprising N- and C-lobes that are embellished by N-lobe insertions and helical extensions at its N and C termini. Structures of *Ph*PINK1 and *Tc*PINK1 dimers revealed that trans-autophosphorylation at a key Ser residue in PINK1 (Ser^202^, Ser^205^, and Ser^228^ in *Ph*PINK1, *Tc*PINK1, and *Hs*PINK1, respectively) activates the kinase (*12*, *13*). Furthermore, structures of phosphorylated *Ph*PINK1, with and without ubiquitin, demonstrated that autophosphorylation at the key Ser residue stabilizes the third N-lobe insertion (insertion-3), enabling PINK1 to bind and phosphorylate ubiquitin (*12*, *20*). *Tc*PINK1 structures have been determined in complex with adenosine 5′-triphosphate (ATP) analogs, which expectedly bind the cleft between the N- and C-lobes (*13*, *22*).

Enhancing mitophagy by pharmacologically increasing PINK1 activity has been considered as a potential strategy to treat PD (*24*, *25*). In a key study, an analog of ATP, kinetin triphosphate (KTP), was reported to be used by PINK1 with greater efficiency than ATP and restored the activity of a PINK1 EOPD mutant (*26*). While KTP itself is impermeable to the cell membrane and therefore of limited therapeutic value, its membrane permeable precursor, kinetin, can be intracellularly metabolized into KTP and appeared to accelerate PINK1/Parkin mitophagy (*26*, *27*). However, it remains unclear whether the PINK1 activating effect of kinetin is mediated through KTP or whether an alternate mechanism is involved (*28*). Regardless, these compounds demonstrate a considerable therapeutic potential, and it is important to understand the molecular basis underlying their activities.

Here, we investigate PINK1’s interaction with the nucleotides adenylyl-imidodiphosphate (AMP-PNP) and adenosine 5′-diphosphate (ADP) and with the reported PINK1 activators KTP and kinetin. We exploit a *Ph*PINK1 dodecamer as a platform to determine nucleotide-bound PINK1 structures by cryo–electron microscopy (cryo-EM). While structures of AMP-PNP–bound and ADP-bound *Ph*PINK1 reveal nucleotide-induced conformational changes in the PINK1 N-lobe, we failed to detect any binding between PINK1 and KTP. Instead, KTP binding is blocked by PINK1’s gatekeeper residue, which is analogous to many other kinases. Mutation of the Met gatekeeper residue to smaller residues enables KTP binding, and mutation to a Gly switches PINK1’s nucleotide preference from ATP to KTP, which inactivates PINK1 in cells. Mutated PINK1 can now be activated by treatment of cells with kinetin and be used as a conditional activator of gatekeeper-mutated PINK1, allowing us to uncouple PINK1 stabilization and PINK1 activity in mitophagy settings.

## RESULTS

### A PhPINK1 dodecamer enables atomic-resolution cryo-EM structures of PINK1 bound to nucleotides

**Fig. 1.**
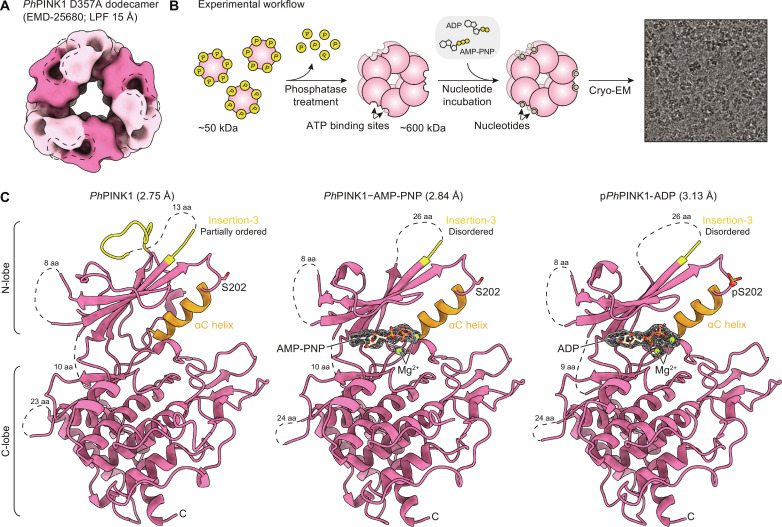
Determining nucleotide-bound *Ph*PINK1 structures. (**A**) A 15-Å low-pass–filtered (LPF) cryo-EM density map of the published *Ph*PINK1 D357A dodecamer [(*12*); EMD-25680]. Individual *Ph*PINK1 monomers are shown in alternating colors. Accessible ATP binding sites are highlighted in the dotted outlines (two binding sites per enclosed outline). (**B**) Workflow to generate the wild-type (WT) *Ph*PINK1 dodecamer for cryo-EM analysis in complex with nucleotides. The illustrative micrograph image is reused from fig. S2C; see there for experimental detail. (**C**) Structures of the nucleotide-free, AMP-PNP–bound, and ADP-bound *Ph*PINK1 dimer (only chain B is shown; see fig. S3 for whole dimers) at 2.75-, 2.84-, and 3.13-Å resolution, respectively. Insertion-3 and the αC helix are colored in yellow and orange, respectively. Density of AMP-PNP, ADP, and Mg^2+^ in the *Ph*PINK1 ATP binding site are shown as a mesh. aa, amino acids. Dotted lines indicate regions lacking cryo-EM density.

We recently reported that a *Ph*PINK1 construct (residues 115 to 575) in its unphosphorylated state could assemble into a homo-dodecameric complex, enabling structural determination of the complex by cryo-EM (*12*). However, these structures of *Ph*PINK1 did not contain nucleotides and were obtained with kinase inactive mutants or had undergone Cys–cross-linking procedures. The arrangement of molecules within the dodecamer (comprising six *Ph*PINK1 dimers) provided key insights into PINK1 activation, and the *Ph*PINK1 dodecamer itself, with open and unobstructed ATP binding sites (Fig. 1A), was the ideal platform to determine whether and how nucleotide binding alters PINK1 conformation. We therefore sought to determine structures of wild-type (WT) *Ph*PINK1, without cross-linking, bound to ADP or the non-hydrolyzable ATP analog AMP-PNP.

Thermal shift assays confirmed that ADP, ATP, and AMP-PNP, each in the presence of Mg^2+^, bind and stabilize monomeric *Ph*PINK1 (fig. S1). To obtain WT *Ph*PINK1 dodecamers for cryo-EM analysis, *Ph*PINK1 was purified from bacteria in its monomeric and autophosphorylated form (*20*). Dephosphorylation using λ-phosphatase (λ-PP) induces oligomerization into dodecamers that could be isolated and purified (fig. S2A; see Materials and Methods). Phos-tag analysis, which resolves proteins according to phosphorylation status, confirmed that *Ph*PINK1 was homogeneously dephosphorylated (fig. S2B), although a single phosphate remains on Thr^305^ due to the adjacent Pro306 that prevents dephosphorylation by λ-PP (figs. S2B and 3C). The *Ph*PINK1 dodecamer was left untreated or was incubated with AMP-PNP/Mg^2+^ or ADP/Mg^2+^, before preparation of cryo-EM grids (Fig. 1B, fig. S2C, and Materials and Methods). Processing in cryoSPARC (*29*) yielded reconstructions of the sixfold symmetric dodecamer, which was subsequently locally refined using a single dimer (asymmetric unit) to resolutions of 2.75, 2.84, and 3.13 Å for the nucleotide-free, AMP-PNP–bound, and ADP-bound *Ph*PINK1 dimers, respectively (Fig. 1C; fig. S2, C and D; and Table 1). Clear density could be observed for the bound nucleotides and Mg^2+^ ions (Fig. 1C and fig. S3, A and B). The resolution of the maps enabled unambiguous modeling of *Ph*PINK1 with each nucleotide.

**Table 1.** Cryo-EM data collection, refinement, and validation statistics. RMS, root mean square. FSC, Fourier shell correlation. MG, magnesium ion. ANP, AMP-PNP nucleotide.

	*Ph*PINK1 dimer	*Ph*PINK1–AMP-PNP dimer	p*Ph*PINK1-ADP dimer
	(EMD-42804)	(EMD-42806)	(EMD-42807)
	(PDB: 8UYF)	(PDB: 8UYH)	(PDB: 8UFI)
**Data collection and processing**			
Magnification	×96,000	×96,000	×96,000
Voltage (kV)	300	300	300
Electron exposure (*e*^−^/Å^2^)	50	50	50
Defocus range (μm)	−0.5 to −1.5	−0.5 to −1.5	−0.5 to −1.5
Pixel size (Å)	0.808	0.808	0.808
Symmetry imposed	*C*1	*C*1	*C*1
Initial particle images (no.)	322,520	490,247	304,506
Final particle images (no.)	1,272,219	114,790	130,515
Map resolution (Å)	2.75	2.84	3.13
FSC threshold	0.143	0.143	0.143
Map resolution range (Å)	1.8 to 19.4	1.7 to 5.1	1.9 to 6.0
			
**Refinement**			
Initial model used (PDB code)	7T4N	7T4N	7T4N
Model resolution (Å)	2.4	2.8	3.1
FSC threshold	0.143	0.143	0.143
Model resolution range (Å)	2.1 to 2.9	2.7 to 3.1	3.0 to 3.3
Map sharpening *B* factor (Å^2^)	0	−63	−101
Model composition			
Non-hydrogen atoms	6460	6036	5980
Protein residues	801	745	738
Ligands	0	MG, 3; ANP, 2	MG, 4; ADP, 2
*B* factors (Å^2^)			
Protein	86.89	78.60	123.55
Ligand	0	87.71	113.33
RMS deviations			
Bond lengths (Å)	0.002	0.003	0.003
Bond angles (°)	0.430	0.534	0.608
Validation			
MolProbity score	1.28	1.29	1.25
Clashscore	4.72	3.66	4.03
Poor rotamers (%)	1.12	1.52	1.08
Ramachandran plot			
Favored (%)	98.84	98.87	97.85
Allowed (%)	1.16	1.13	2.15
Disallowed (%)	0.00	0.00	0.00

### Nucleotides induce conformational changes in the PINK1 N-lobe

**Fig. 2.**
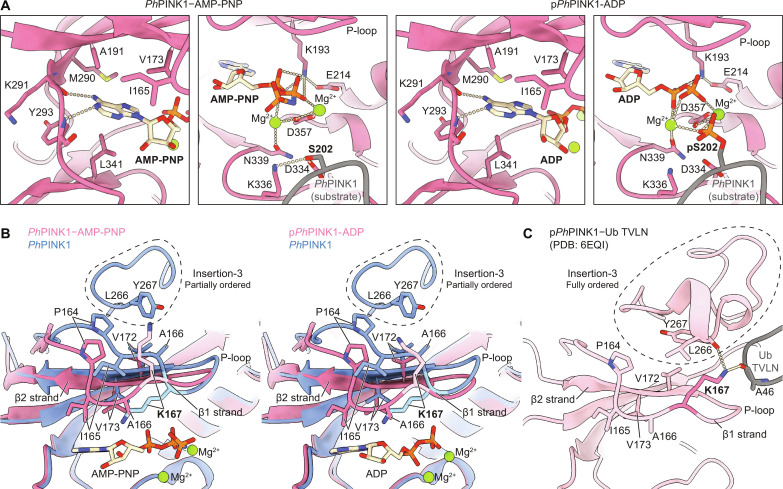
*Ph*PINK1-nucleotide interactions. (**A**) Details of the interaction of AMP-PNP and ADP with the ATP binding site of *Ph*PINK1 (chain B of each dimer). Interacting residues are shown as sticks, and polar interactions are shown as dotted lines. The interaction with (phosphorylated) Ser^202^ of chain A (gray) is also shown. (**B**) Superimpositions of the N-lobe of AMP-PNP–bound and ADP-bound *Ph*PINK1 with nucleotide-free *Ph*PINK1, revealing differences in conformation of the P-loop and insertion-3 (highlighted in the dotted outlines). Lys^167^ is highlighted in a lighter shade. (**C**) The N-lobe of the published phosphorylated and ubiquitin-bound *Ph*PINK1 complex [Ub TVLN, ubiquitin T66V L67N mutant; PDB: 6EQI; (*20*)], in the same orientation as in (B). Lys^167^ (highlighted in a darker shade) interacts with both the ordered insertion-3 and ubiquitin (gray).

The newly generated *Ph*PINK1 structures revealed fresh insights into the interaction of nucleotides with PINK1 and unveiled notable conformational changes that couple nucleotide binding to previously observed conformational changes. The nucleotide-free WT *Ph*PINK1 dimer was virtually indistinguishable from the nucleotide-free *Ph*PINK1 D357A dimer that we determined previously (*12*). Both AMP-PNP and ADP bind the ATP binding site of *Ph*PINK1 in the anticipated nucleotide binding mode and in a similar manner to *Tc*PINK1 (Fig. 2A and fig. S3D) (*13*, *22*). Hydrophobic residues stemming from N- and C-lobes encapsulate the adenine and ribose groups (Fig. 2A). Two hydrogen bonds are made via the N^1^ and N^6^ nitrogens of adenine, which contact the backbone of Tyr^293^ and Lys^291^, respectively, of the kinase hinge region (Fig. 2A). The phosphate groups of AMP-PNP and ADP extend toward the phosphoryl transfer center of the kinase and only indirectly bind the so-called P-loop (an extended Gly-rich β-hairpin loop including the β1 and β2 strands) of the N-lobe (Fig. 2A). The phosphates are held in position by a series of electrostatic interactions with Lys^193^ of the kinase “VAIK” motif (LAVK in *Ph*PINK1) and two Mg^2+^ ions, which themselves are positioned by several polar residues that include Asp^357^ of the kinase “DFG” motif (Fig. 2A).

Comparison of the nucleotide-free and nucleotide-bound states of *Ph*PINK1 revealed prominent reorganization of the kinase P-loop upon nucleotide binding, which affects insertion-3 that eventually forms the ubiquitin binding site at the kinase N-lobe (Fig. 2B). In nucleotide-free (and unphosphorylated) *Ph*PINK1, insertion-3 is only partially visible, but the ordered residues interact with and shield an otherwise exposed hydrophobic patch on the N-lobe, including Pro^164^, Ala^166^, and Val^172^ of the P-loop (Fig. 2B and fig. S4A). This partially ordered conformation of insertion-3 is structurally incompatible with the fully ordered insertion-3 formed after PINK1 N-lobe phosphorylation that becomes the binding site for ubiquitin and Ubl substrates (Fig. 2C) (*12*, *20*). Binding of AMP-PNP or ADP to unphosphorylated *Ph*PINK1 causes the P-loop to clamp onto the nucleotide. P-loop movement (by ~6 Å between the Cα’s of Ile^165^) involves multiple residues, including Val^173^ of the β2 strand that forms part of the catalytic spine (C spine), which is completed upon interaction with the nucleotide (Fig. 2B) (*30*).

Two residues in the β1 strand, Ala^166^ and Lys^167^, flip such that Ala^166^ points its side chain into the ATP binding site, and Lys^167^ points away from the ATP binding site (Fig. 2B). As a net result, clamping down of the P-loop generates additional space for insertion-3 to fold, although without phosphorylation, insertion-3 remains disordered, and several hydrophobic side chains on the N-lobe, including the P-loop, are exposed (Fig. 2B and fig. S4A). Also important is the observed flip in Lys^167^, which only in the nucleotide-bound or phosphorylated state of PINK1 can form interactions with both the ordered insertion-3, as well as with the substrate ubiquitin/Ubl (Fig. 2C) (*12*, *20*).

Together, these observations suggest an unexpected coupling between PINK1 nucleotide binding and the conformation of ubiquitin-binding insertion-3, via conformational rearrangements of the kinase P-loop. Given that phosphorylated *Ph*PINK1 in its nucleotide-free state adopts both active and inactive conformations (*12*), it is tempting to speculate that ATP, in addition to its role as a phosphate donor, may contribute to stabilizing PINK1 in its active and ubiquitin binding-competent state. On the basis of conservation, this may be similar in human PINK1 (figs. S4B and S5; see Discussion). Intrinsic protein dynamics, confined localized changes, and low overall binding affinities make such conformational transitions difficult to assess biochemically.

### Structures reveal a post-catalytic dimerized state of PINK1

Unexpectedly, we find that ADP-bound *Ph*PINK1 is autophosphorylated at Ser^202^, most likely due to contaminating ATP in the ADP stock that we used (Fig. 2A and fig. S3, E to G). This additional phosphate group interacts with both Mg^2+^ ions in the active site and is positioned ~5 Å from the β-phosphate of the bound ADP (Fig. 2A and fig. S3G). The phosphorylated *Ph*PINK1-ADP dimer represents the post-catalytic state of PINK1 immediately following phosphoryl transfer but before dimer dissociation [see (*12*)]. In this situation, *Ph*PINK1 molecules within the dimer remain entirely in their inactive conformation, with an extended αC helix and disordered insertion-3, contrasting the conformational shift observed in our previous structure of phosphorylated and cross-linked *Ph*PINK1 dimer (*12*). A possible reason why the conformational change is not observed in this scenario could be that the high concentration of ADP in the sample sits snugly with phosphorylated Ser^202^ (pSer^202^) in the active site of a stable kinase dimer composition (fig. S3G).

### PINK1 cannot use KTP due to a clash with a gatekeeper residue

**Fig. 3.**
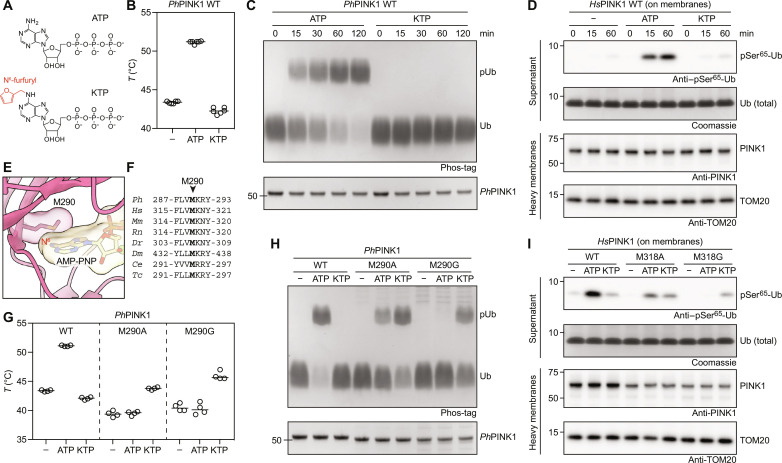
The PINK1 gatekeeper residue prevents KTP binding. (**A**) Chemical structures of ATP and KTP. The N^6^-furfuryl group of KTP is highlighted in red. (**B**) Melting temperatures of WT *Ph*PINK1 (residues 115 to 575) in the presence of ATP or KTP. Experiment was performed three times in technical duplicates. (**C**) Time course ubiquitin phosphorylation assay using WT *Ph*PINK1 in the presence of ATP or KTP and analyzed by Phos-tag SDS–polyacrylamide gel electrophoresis (PAGE). Experiment was performed in triplicate. (**D**) Time course ubiquitin phosphorylation assay using *Hs*PINK1-containing heavy membranes from OA-treated HeLa cells in the presence of ATP or KTP (see Materials and Methods) and analyzed by Western blotting. Experiment was performed in triplicate. (**E**) The ATP binding site of AMP-PNP–bound *Ph*PINK1, revealing that the gatekeeper Met^290^ would sterically block an N^6^-modified ATP analog from binding *Ph*PINK1. (**F**) Conservation of the PINK1 gatekeeper residue. *Ph*, *Pediculus humanus corporis*; *Hs*, *Homo sapiens*; *Mm*, *Mus musculus*; *Rn*, *Rattus norvegicus*, *Dr*, *Danio rerio*; *Dm*, *Drosophila melanogaster*; *Ce*, *Caenorhabditis elegans*; *Tc*, *Tribolium castaneum*. (**G**) Melting temperatures of *Ph*PINK1 WT and gatekeeper mutants M290A and M290G in the presence of ATP or KTP. Experiment was performed two times in technical duplicates, while M290A in the absence of nucleotide was performed three times in technical duplicates (see fig. S7). (**H**) Ubiquitin phosphorylation assay using *Ph*PINK1 WT and gatekeeper mutants M290A and M290G in the presence of ATP or KTP for 2 hours and analyzed by Phos-tag SDS-PAGE. Experiment was performed in triplicate. (**I**) Ubiquitin phosphorylation assay using *Hs*PINK1-containing heavy membranes from OA-treated HeLa cells in the presence of ATP or KTP for 2 hours (see Materials and Methods) and analyzed by Western blotting. Experiment was performed in triplicate.

We next attempted to investigate how the reported PINK1 activator KTP (Fig. 3A) interacts with PINK1 (*26*). Using thermal shift binding assays, we first tested whether KTP stabilizes *Ph*PINK1 and observed that, unlike ATP, KTP does not stabilize *Ph*PINK1 (Fig. 3B and fig. S6C). Next, in vitro ubiquitin phosphorylation assays showed that *Ph*PINK1 could not phosphorylate ubiquitin when KTP was the sole nucleotide source (Fig. 3C). Given that the previous study reporting PINK1 activity with KTP was based on human PINK1 (*26*), we tested whether sequence differences between *Ph*PINK1 and *Hs*PINK1 (~40% kinase domain identity) may account for the inability of KTP to work with *Ph*PINK1. To minimize these differences, we mutated the three differing residues within the *Ph*PINK1 ATP binding site to *Hs*PINK1 equivalent residues (V247I, M249V, and T356A), generating a humanized version of *Ph*PINK1 (fig. S6A). Despite now harboring a *Hs*PINK1-like ATP binding site, the *Ph*PINK1 V247I/M249V/T356A triple mutant was neither stabilized nor showed ubiquitin kinase activity with KTP (fig. S6, B to D).

Next, we investigated *Hs*PINK1 activity, using heavy membranes from oligomycin and antimycon (OA)-treated HeLa *PINK1*^−/−^ cells transiently expressing WT *Hs*PINK1 that were incubated with recombinant ubiquitin in the presence of either ATP or KTP (see Materials and Methods). Incubation with ATP resulted in phosphorylation of ubiquitin at Ser^65^, as detected by a pSer^65^ ubiquitin antibody (Fig. 3D). While KTP incubation resulted in a faint phospho-ubiquitin band, a similarly faint band was also visible in the absence of any added nucleotide, indicating the presence of residual ATP in the crude heavy membrane preparation used for the assay (Fig. 3D). Together, these results indicate that PINK1 is unable to use KTP as a phosphate donor.

Why was PINK1 unable to use KTP in our experiments? KTP is an analog of ATP, defined by an additional furfuryl group covalently attached to the N^6^ of the adenine ring (Fig. 3A). N^6^-substituted ATP analogs with bulky groups such as furfuryl typically cannot be accommodated by protein kinases due to a clash with a so-called gatekeeper residue at the back of the ATP binding site (*31*). It is possible that the conserved gatekeeper residue of PINK1 (Met^290^ in *Ph*PINK1 and Met^318^ in *Hs*PINK1) obstructs KTP (Fig. 3, E and F). Mutation of Met^290^ in *Ph*PINK1 to smaller Ala or Gly residues while affecting recombinant protein yield and stability (fig. S7) enabled the kinase to be stabilized by KTP and to use KTP as a phosphate donor in ubiquitin phosphorylation experiments (Fig. 3, G and H). These results were mirrored in *Hs*PINK1 M318A and M318G mutants on mitochondria enriched from OA-treated HeLa cells (Fig. 3I). While mutant kinases were expressed at lower levels compared to WT PINK1 (Fig. 3I), the Gly mutation greatly diminished PINK1’s ability to use ATP, consistent with the idea that the gatekeeper is also important for ATP binding (Fig. 3, H and I). However, both mutants were able to use KTP instead of ATP to generate phospho-ubiquitin.

Together, our results show that contrary to what has been suggested (*26*, *32*, *33*) PINK1 may not bind KTP or use it as a preferred phosphate donor in a direct ATP-competitive fashion. We could enable PINK1 to use KTP by mutating the gatekeeper Met residue in the PINK1 ATP binding site in insect and human PINK1. Switching PINK1’s nucleotide preference from ATP to KTP was next exploited to decouple PINK1 stabilization from PINK1 activity.

### Kinetin activates human PINK1 gatekeeper mutants in cells

**Fig. 4.**
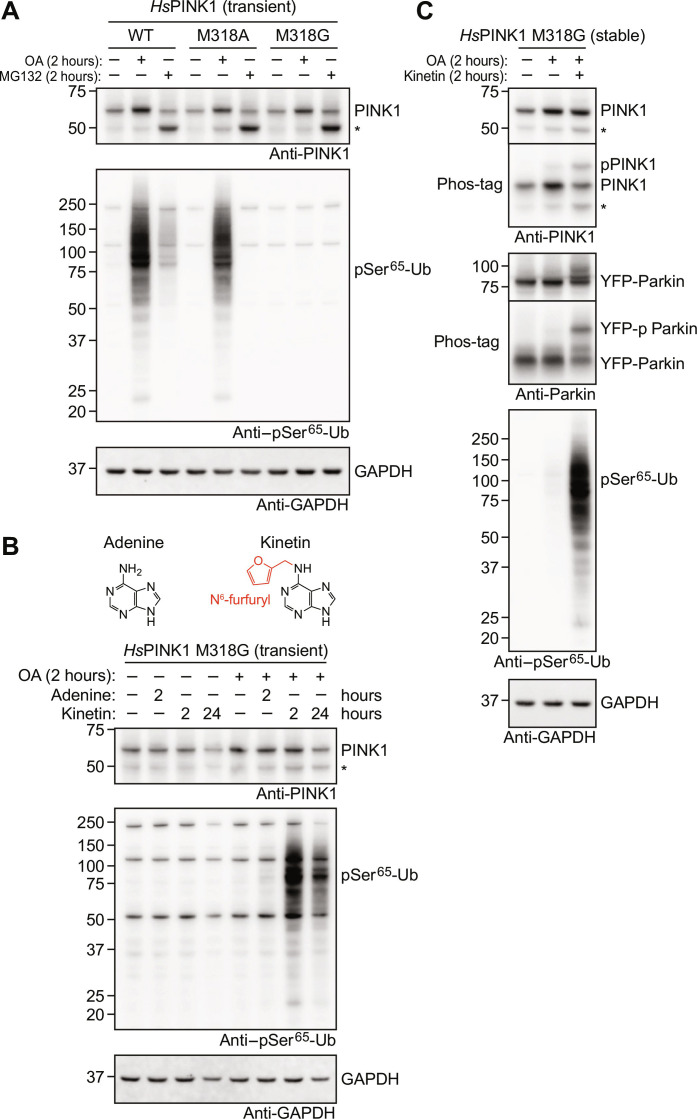
Kinetin activates *Hs*PINK1 M318G in cells. (**A**) HeLa *PINK1*^−/−^ YFP-Parkin cells transiently expressing *Hs*PINK1 WT, M318A, and M318G were treated with OA or MG132 for 2 hours and then immunoblotted for PINK1 and phospho-Ser^65^-ubiquitin (pSer^65^-Ub). *Hs*PINK1 M318G is stabilized upon OA treatment but is unable to generate phospho-ubiquitin. Experiment was performed in triplicate. (**B**) HeLa *PINK1*^−/−^ YFP-Parkin cells transiently expressing *Hs*PINK1 M318G were treated with 200 μM adenine for 2 hours or 200 μM kinetin for 2 or 24 hours. OA was added 2 hours before lysis. Immunoblotting revealed that the addition of kinetin cotreatment with OA activates *Hs*PINK1 M318G and leads to ubiquitin phosphorylation. Experiment was performed in triplicate. (**C**) HeLa *PINK1*^−/−^ YFP-Parkin cells stably expressing *Hs*PINK1 M318G treated for 2 hours with OA alone or in the presence of 200 μM kinetin. As in (B), immunoblotting revealed that kinetin activates ubiquitin phosphorylation. Additional Phos-tag analysis shows induction of PINK1 autophosphorylation and Parkin phosphorylation. Experiment was performed in triplicate. An asterisk in (A) to (C) indicates the 52-kDa PARL-cleaved PINK1. GAPDH, glyceraldehyde-3-phosphate dehydrogenase.

We assessed the impact of the gatekeeper mutants M318A and M318G on *Hs*PINK1 function in intact HeLa *PINK1*^−/−^ cells expressing YFP-Parkin that were transiently transfected with constructs encoding *Hs*PINK1 variants. *Hs*PINK1 WT accumulated in response to OA and generated phospho-ubiquitin, as expected (Fig. 4A). Accumulation of PINK1 was also observed for *Hs*PINK1 M318A and M318G mutants, and proteasome inhibition induced similar accumulation of the 52-kDa presenilin-associated rhomboid-like protein (PARL)-cleaved PINK1 fragment, indicating that basal turnover of PINK1 variants was unimpaired (Fig. 4A). While a *Hs*PINK1 M318A mutant phosphorylated ubiquitin at slightly reduced levels compared with WT, the M318G mutant generated phospho-ubiquitin at barely detectable levels (Fig. 4A), consistent with in vitro experiments (Fig. 3H).

Given the M318G mutant’s preference for KTP over ATP, we wondered whether its inactivity in cells can be overcome by supplying KTP. However, direct KTP treatment is unfeasible as ATP analogs are unable to cross the cell membrane. Instead, the KTP precursor, kinetin, is membrane permeable and has been shown to be intracellularly metabolized into KTP (*26*). We therefore attempted to activate *Hs*PINK1 M318G in cells with kinetin. Treatment with OA alone for 2 hours did not activate the M318G mutant, but cotreatment with 200 μM kinetin, but not adenine, led to substantial generation of phospho-ubiquitin (Fig. 4B). Increasing the duration of kinetin treatment to 24 hours did not increase the level of phospho-ubiquitin but instead hampered cell growth and/or survival (Fig. 4B). Kinetin did not enhance the activity of *Hs*PINK1 WT, suggesting the effect of kinetin is specific to *Hs*PINK1 M318G (fig. S8). These results are consistent with kinetin undergoing intracellular conversion into KTP, which then acts as a phosphate donor specifically for the *Hs*PINK1 M318G gatekeeper mutant.

We also generated cells stably expressing *Hs*PINK1 M318G. As was observed in transient expression experiments, stably expressed *Hs*PINK1 M318G accumulated in response to OA treatment and generated barely detectable levels of phospho-ubiquitin unless cotreated with kinetin (Fig. 4C). Phos-tag analysis further revealed that kinetin cotreatment increased PINK1 autophosphorylation and Parkin phosphorylation (Fig. 4C).

### Kinetin-activated PINK1 M318G recruits Parkin to mitochondria

**Fig. 5.**
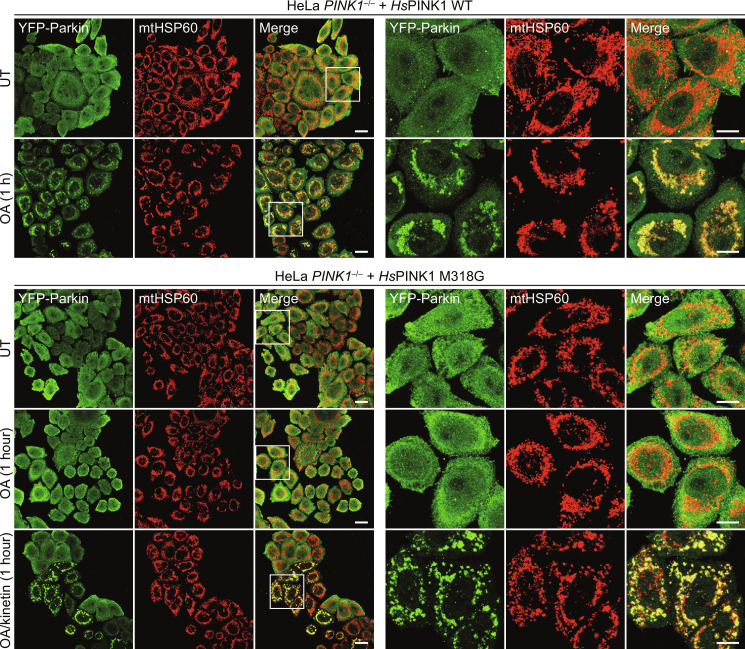
Kinetin-activated *Hs*PINK1 M318G induces Parkin translocation. YFP-Parkin translocation in fixed HeLa *PINK1*^−/−^ cells stably expressing *Hs*PINK1 WT or M318G and immunostained for mtHSP60 (mitochondrial marker). Cells were treated with either OA alone or in combination of 200 μM kinetin for 1 hour before fixing and staining. While YFP-Parkin translocation was induced with OA alone in cells expressing *Hs*PINK1 WT, translocation in *Hs*PINK1 M318G-expressing cells was only induced when cotreated with kinetin. However, the M318G mutant is not completely inactive; when cells were treated with OA alone for 2 hours, YFP-Parkin translocation was observable (see fig. S9). Scale bars, 20 μm (left three columns) and 10 μm (right three columns). White boxes (left) depict the zoomed-in area. Experiment was performed in duplicate with fig. S9, and representative images are shown. YFP, yellow fluorescent protein.

Because Parkin must be in proximity to OMM-stabilized PINK1 to become phosphorylated, our results suggested that kinetin-activated *Hs*PINK1 M318G could induce translocation of Parkin to mitochondria and trigger mitochondrial clearance via mitophagy. To test this, we performed immunofluorescence using HeLa *PINK1*^−/−^ cells expressing YFP-Parkin and *Hs*PINK1 to assess YFP-Parkin translocation to mitochondria. As anticipated, by the 1-hour time point of OA treatment, WT *Hs*PINK1 had robustly recruited YFP-Parkin to mitochondria (Fig. 5). In contrast, *Hs*PINK1 M318G did not recruit Parkin after 1 hour of OA treatment; however, when cotreated with kinetin, robust recruitment was observed at 1 hour of OA/kinetin treatment (Fig. 5).

Together, the results indicate that *Hs*PINK1 M318G is functionally compromised to induce mitophagy in normal cells but now relies on a distinct nucleotide source, KTP, which is generated in situ from kinetin. We have hence established an orthogonal system to induce mitophagy, in which PINK1 stabilization and PINK1 activation are uncoupled.

## DISCUSSION

In the first part of this study, we exploited established cryo-EM workflows to characterize the interaction of *Ph*PINK1 with nucleotides. Our structures reveal localized conformational changes that occur upon nucleotide binding (Fig. 2). Crystal structures of nucleotide-free and AMP-PNP–bound *Tc*PINK1 have been reported and compared previously (*13*, *21*, *22*), with a focus on understanding dimer formation and autophosphorylation reactions. We used cryo-EM to directly compare the nucleotide-bound and unbound states of *Ph*PINK1, revealing conformational changes in the kinase P-loop and insertion-3 (fig. S5). Before nucleotide binding, insertion-3 shields a hydrophobic patch in the N-lobe, likely to maintain protein stability and prevent nonspecific interactions with other proteins before the activation of PINK1. Nucleotide binding is relayed via the flexible P-loop and opens the N-lobe to accommodate insertion-3; however, without phosphorylation, insertion-3 remains disordered. Such exposed state of PINK1 would be short-lived because subsequent trans-autophosphorylation of PINK1 would cause insertion-3 to reconfigure into its activated and ubiquitin binding conformation, thereby reshielding the hydrophobic patch (*12*, *13*, *20*). Furthermore, following autophosphorylation, insertion-3 remains dynamic and exchanges between folded and unfolded states, as we have previously resolved using three-dimensional (3D) variability analysis (*12*). Therefore, it is possible that ATP binding, in addition to it serving as a phosphate donor, helps to further stabilize insertion-3 in its folded and ubiquitin binding conformation, increasing the efficiency of ubiquitin binding and phosphorylation.

The ease with which the nucleotide-bound *Ph*PINK1 structures could be solved means that the *Ph*PINK1 dodecamer could now be used as a platform to understand the binding mechanism of other kinase-interacting molecules, such as PINK1-activating compounds. We had envisaged to use our platform to understand how KTP interacts with PINK1; however, we could not biochemically reproduce the reported PINK1-binding and activating properties of KTP (*26*). With our detailed structural understanding of PINK1, we define exactly why KTP cannot interact with PINK1: The PINK1 gatekeeper residue prevents the enlarged nucleobase to bind in the ATP binding pocket due to a steric clash between the KTP furfuryl group and the gatekeeper Met side chain. Mutation of the gatekeeper to the smaller residue such as Ala and Gly enables KTP to bind and activate PINK1. These results are consistent with numerous reports of kinases that require a gatekeeper mutation to use KTP (and KTP analogs such as KTPγS) as their phosphate source (*34*–*39*). A proteome-wide, mass-spectrometry based study identified 26 putative KTP-binding kinases out of the 218 detected kinases in cell lysates (*40*). No endogenous human kinase bound KTP preferentially and even kinases that seemingly bound to KTP did not efficiently use KTP as a phosphate donor (*40*). That study did not analyze PINK1, possibly because of its very low abundance in unstimulated cells. Yet, our biochemical data presented here strongly suggest that WT PINK1 is unable to bind KTP or use it as a phosphate donor, let alone in a preferential manner.

The KTP precursor kinetin and its derivatives have been thought to activate PINK1 in cells by undergoing intracellular conversion into KTP to function as a phosphate donor (*26*, *27*, *33*). Our data now indicate that the underlying mechanism behind kinetin-induced PINK1 activation, which has been reproduced by others, is unlikely to occur via KTP conversion and the provision of an improved nucleotide source to PINK1. A recent study reported that a divergent kinetin analog, MTK458, cannot be converted into a triphosphate form yet was able to activate PINK1 (*28*). How MTK458 acts on PINK1 will require further analysis and may also illuminate the role of kinetin in PINK1 biology.

Kinetin or MTK458 appear to work more efficiently in combination with subthreshold doses of depolarizing agents that are insufficient to trigger phospho-ubiquitin generation and mitophagy on their own (*27*, *28*). We used kinetin in combination with typical mitophagy-inducing doses of the depolarizing agent OA but did not detect kinetin-mediated amplification of *Hs*PINK1 activity. Instead, we saw a remarkable sensitization to kinetin-mediated activation when the M318G gatekeeper mutation was introduced. The specificity of kinetin toward the M318G mutant strongly indicates that the mechanism underlying PINK1 activation in our case is via the conversion of kinetin into KTP, which then acts directly on PINK1 M318G via its enlarged ATP binding site.

Because *Hs*PINK1 M318G accumulates on mitochondria upon depolarization yet remains almost completely inactive, kinetin may be used to specifically activate pre-accumulated *Hs*PINK1 M318G, essentially decoupling PINK1 activity from stabilization of the protein through mitochondrial depolarization. A similar goal has been achieved previously using a temperature-sensitive mutant of PINK1 that becomes active when the temperature is lowered from 37° to 22°C (*41*). Our strategy, while not needing a temperature shift, requires the initial conversion of kinetin into KTP. On the basis of our immunofluorescence imaging experiments, conversion to KTP produces an effect on Parkin recruitment within 1 hour. If a more rapid conversion of kinetin is required, it may be possible to use kinetin riboside derivatives that reduce the number of conversion steps into KTP (*33*). We note that *Hs*PINK1 M318G is not a kinase inactive mutant because it can function with ATP when expressed at high levels and subjected to extended OA treatments. Therefore, *Hs*PINK1 M318G expression should be titrated to a level that induces minimal OA-induced Parkin recruitment and mitophagy while maintaining robust activation of kinase activity by kinetin.

The second kinase gatekeeper mutant that we used, *Hs*PINK1 M318A, is active with both ATP and KTP and remains functional in the absence of kinetin. This mutant has been used previously for the purpose of sensitizing PINK1 to inhibition by PP1 analogs (such as small molecule compounds 1-NA-PP1 and 1-NM-PP1) that were specifically designed to inhibit gatekeeper-mutated kinases (*42*, *43*). It is likely that *Hs*PINK1 M318G may also be inhibited by PP1 analogs, given that PP1 analogs inhibit many kinases harboring a Gly gatekeeper mutation (*42*). Therefore, kinetin and PP1 analogs, in combination with the *Hs*PINK1 M318A and M318G gatekeeper mutants, may comprise a powerful chemical genetics toolkit for the activation or inhibition of PINK1 activity in cells in an experimentally controlled manner.

## MATERIALS AND METHODS

### Molecular cloning

DNA encoding *Ph*PINK1 (residues 115 to 575), codon optimized for expression in *Escherichia coli*, was inserted in between the Kpn I and Hind III sites of the pOPINK vector (*44*) using the In-Fusion HD Cloning Kit (Takara). The pOPINK vector incorporates an N-terminal GST tag and a 3C protease cleavage site into the *Ph*PINK1 construct. *Ph*PINK1 mutants were generated using the Q5 Site-Directed Mutagenesis Kit [New England Biolabs (NEB)] using the following primers: *Ph*PINK1_V247I_M249V_F (TCCGAATATTaTTCGTgTGTATAGCGTTTTTGCAGATCGTATTC), *Ph*PINK1_V247I_M249V_R (TGCGGAGGCAGACGAATT), *Ph*PINK1_T356A_F (CATTGTTATTgCCGATTTTGGTTG), *Ph*PINK1_T356A_R (GTCGGATATGCATCACCTTC), *Ph*PINK1_M290A_F (GTTTCTGGTTgcaAAACGTTATGATTGC), *Ph*PINK1_M290G_F (GTTTCTGGTTggtAAACGTTATGATTGCAC), and *Ph*PINK1_M290_R (AGGCTCATATTACGACCAC).

### Protein expression and purification

All *Ph*PINK1 (residues 115 to 575) constructs and λ-PP were expressed in *E. coli* Rosetta2 (DE3) pLacI cells (Novagen) and purified as described previously (*12*). To generate the WT *Ph*PINK1 dodecamer for cryo-EM analysis, ~11 mg of purified WT *Ph*PINK1 (residues 115 to 575), at a concentration of 15 μM, was dephosphorylated with 7.5 μM λ-PP in 25 mM tris (pH 8.5), 500 mM NaCl, 2 mM MnCl_2_, 5% (v/v) glycerol, 10 mM dithiothreitol (DTT) for 24 hours at 4°C. To promote *Ph*PINK1 oligomerization, the concentration of NaCl was reduced by buffer exchange into 25 mM tris (pH 8.5), 150 mM NaCl, and 10 mM DTT using a HiPrep 26/10 Desalting column (Cytiva) and then incubated for 3 hours at 4°C. The resulting *Ph*PINK1 dodecamer was purified by size exclusion chromatography (SEC) using a HiLoad 26/600 Superdex 200 pg column (Cytiva) in 25 mM tris (pH 8.5), 150 mM NaCl, and 10 mM DTT, and fractions corresponding to the dodecamer were pooled. Anion exchange chromatography was used to concentrate the dodecamer. Pooled fractions from SEC were applied to a Mono Q 5/50 GL column (Cytiva) in 25 mM tris (pH 8.5), 50 mM NaCl, and 10 mM DTT and eluted with a 0 to 50% linear gradient of 25 mM tris (pH 8.5), 1 M NaCl, and 10 mM DTT over 20 column volumes. The *Ph*PINK1 dodecamer eluted at approximately 250 mM NaCl. The fraction containing the highest concentration of *Ph*PINK1 (3.4 mg/ml) was diluted with 25 mM tris (pH 8.5) and 10 mM DTT to achieve a 150 mM NaCl concentration, resulting in a final *Ph*PINK1 concentration of 1.9 mg/ml. The protein was then flash-frozen in liquid nitrogen and stored at −80°C.

### Cryo-EM sample preparation and data collection

To generate the *Ph*PINK1-nucleotide complexes, purified *Ph*PINK1 dodecamer (1.9 mg/ml) was incubated with 10 mM AMP-PNP or ADP and 10 mM MgCl_2_ for 10 to 25 min before vitrification. The nucleotide-free or nucleotide-bound dodecamers were dispensed onto glow discharged UltrAuFoil (Quantifoil GmbH, Germany) R1.2/1.3 holey specimen support (“grid”) at 100% humidity, 4°C, and blotted for 4 s (nominal blot force of −1). Grids were then plunge frozen in liquefied ethane using a Vitrobot Mark IV (Thermo Fisher Scientific). Data were collected using a Titan Krios G4 microscope equipped with a Falcon 4 direct electron detector (Thermo Fisher Scientific) using a nominal magnification of ×96,000, corresponding to a pixel size at the detector of 0.808 Å.A total of 2746, 3094, and 2923 movies were captured for the nucleotide-free, AMP-PNP–bound, and ADP-bound *Ph*PINK1 datasets, respectively.

### Cryo-EM refinement and model building

Cryo-EM processing was performed in cryoSPARC (v4.2.1) (*29*). All *Ph*PINK1 datasets were processed using a similar workflow, detailed in fig. S2C. All movies were patch-motion–corrected, and contrast transfer function (CTF) parameters were estimated using the patch CTF job. Templates were generated from blob picker performed on the p*Ph*PINK1-ADP dataset and used to pick particles from all datasets using template picker. Particles were extracted and binned (downsampled) two times, and 2D classification was performed. Classes with any PINK1-like features were selected as “good” particles, and ab initio reconstruction was performed to generate an initial reconstruction of the *Ph*PINK1 dodecamer. A second subset of classes containing particles of indistinct shapes was selected as “junk” particles, and ab initio reconstruction was performed to generate a volume for subsequent heterogeneous refinement. Three rounds of heterogeneous refinement were performed against the good and junk maps in *C*_1_ to remove bad particles from the dataset. Reconstruction of the dodecamer was performed using homogeneous refinement in *D*_3_. A mask of the dimer was generated by zoning the map from homogenous refinement against a model of the *Ph*PINK1 D357A dimer [Protein Data Bank (PDB): 7T4N] using UCSF ChimeraX (*12*, *45*), dilated, and soft-padded. Particles were symmetry expanded in *D*_3_, recentered, and locally refined using the dimer mask. For the AMP-PNP–bound and ADP-bound *Ph*PINK1 datasets, signal subtraction was performed before local refinement. 3D variability analysis was performed solving for three modes, and the particles were clustered to give ~100,000 particles per cluster. Particles corresponding to the most homogeneous and complete cluster were then locally refined to give the final dimer reconstruction.

Model building was performed in Coot (v0.9.8.7) (*46*), and refinement was performed using real-space refinement in Phenix (v1.20.1-4487) (*47*). The *Ph*PINK1 D357A dimer (PDB: 7T4N) (*12*) was used as the initial model and was docked into the density of the nucleotide-free *Ph*PINK1 dimer using UCSF ChimeraX (v1.6.1) (*45*). After a round of model building in Coot and refinement in Phenix, the model was docked in the densities of the *Ph*PINK1–AMP-PNP and p*Ph*PINK1-ADP dimers, and nucleotides and Mg^2+^ ions were fitted into the densities. Model building and refinement were then performed on all three models. Regions with disordered/ambiguous densities were not modeled. Cryo-EM data collection and refinement statistics are provided in Table 1.

### Thermal shift assays

Thermal shift assays were carried out using 4 μM *Ph*PINK1 (residues 115 to 575) and 5× SYPRO Orange Protein Gel Stain (Invitrogen) in 25 mM tris (pH 8.5), 150 mM NaCl, and 10 mM DTT, in the presence of 5 mM ADP (Sigma-Aldrich), AMP-PNP (Sigma-Aldrich or Roche), ATP (Sigma-Aldrich) or KTP (Biolog). MgCl_2_ (10 mM) was included, unless indicated otherwise. Melt curves were measured on a Rotor-Gene Q (Qiagen) with a temperature ramp of 25° to 80°C at 1°C/min and analyzed using the Rotor-Gene Q Series Software (v2.3.1). Graphs were generated in GraphPad Prism (v9.5.1).

### Ubiquitin phosphorylation assays

Ubiquitin phosphorylation assays were carried using 1.5 μM *Ph*PINK1 (residues 115 to 575) and 15 μM ubiquitin in 25 mM tris (pH 7.4), 150 mM NaCl, 10 mM MgCl_2_, and 1 mM DTT. Reactions were initiated by the addition of 1 mM ATP (Sigma-Aldrich) or KTP (Biolog) and incubated at 22°C for 2 hours or as indicated. Reactions were quenched in SDS sample buffer [66 mM tris (pH 6.8), 2% (w/v) SDS, 10% (v/v) glycerol, 0.005% (w/v) bromophenol blue, and 50 mM DTT)], and samples were run on 17.5% Phos-tag gels [containing 50 μM Phos-tag Acrylamide AAL-107 (Wako) and 100 μM MnCl_2_)] and NuPAGE 4 to 12% Bis-Tris gels (Invitrogen). All gels were stained with InstantBlue Coomassie Protein Stain (Abcam).

### Cell culture and constructs

HeLa *PINK1*^−/−^ cells were as described previously (*48*). All HeLa cell lines (RRID:CVCL_0030) were cultured at 37°C, 5% CO_2_, in Dulbecco’s modified Eagle’s medium supplemented with 10% (v/v) fetal bovine serum (Bovogen Biologicals) and penicillin-streptomycin. Cells were routinely checked for mycoplasma contamination using the MycoAlert Mycoplasma Detection Kit (Lonza). For transient expression, DNA encoding *Hs*PINK1 was inserted into the Bam HI site of pcDNA5/FRT/TO CMVd3 vector [pcDNA5^d3^; see (*12*)] using the In-Fusion HD Cloning Kit (Takara). For stable expression, the *Hs*PINK1 sequence was inserted in between the Bam HI and Nhe I sites of the pFU MCS SV40 Puro lentiviral vector (pFUP). Mutagenesis was performed using the Q5 Site-Directed Mutagenesis Kit (NEB) using the following primers: *Hs*PINK1_M318A_F (GTTTCTCGTGgccAAGAACTACCCTTGCACC), *Hs*PINK1_M318G_F (GTTTCTCGTGggcAAGAACTACCCTTGCACC), and *Hs*PINK1_M318_R (AGGGTTCTGCCGTGTCCC).

### Generation of stable cell lines

HeLa *PINK1*^−/−^ cells stably expressing YFP-Parkin and *Hs*PINK1 were generated by sequential introduction of YFP-Parkin followed by *Hs*PINK1 into HeLa *PINK1*^−/−^ cells. YFP-Parkin was introduced using retroviral transduction with the pBMN-YFP-Parkin plasmid (gift from R. Youle; Addgene plasmid, 59416), followed by fluorescence sorting. *Hs*PINK1 WT and the M318G mutant were introduced using lentiviral transduction with pFUP-*Hs*PINK1 plasmids, followed by selection with puromycin.

### Transient transfection and Western blotting

Cells for transient transfection were seeded in six-well plates 24 to 48 hours before transfection. Transient transfections were performed with 1.5 μg of pcDNA5^d3^-*Hs*PINK1 plasmids using the Lipofectamine 3000 Transfection Reagent (Invitrogen), and transfected cells were allowed to grow for 24 hours before harvesting. Cell lines stably expressing *Hs*PINK1 were seeded in six-well plates 48 hours before harvesting. To depolarize mitochondria to induce PINK1 stabilization, cells were treated with 10 μM oligomycin and 4 μM antimycin A (OA) for the indicated times. Adenine and kinetin treatments were performed at 200 μM for the indicated times, and MG132 treatments were performed at 10 μM for 2 hours. Cell lysates were prepared directly in SDS sample buffer and then separated on NuPAGE 4 to 12% Bis-Tris gels (Invitrogen) or 7.5% Phos-tag gels [containing 50 μM Phos-tag Acrylamide AAL-107 (Wako) and 100 μM MnCl_2_]. Phos-tag gels were washed three times for 10 min in 10 mM EDTA and 10 min in water before transfer. Protein transfer was carried out using the Trans-Blot Turbo Transfer System (Bio-Rad) onto polyvinylidene difluoride membranes. Membranes were then blocked in 5% (w/v) skim milk powder in tris-buffered saline containing 0.1% Tween 20 (TBS-T) and incubated with primary antibodies in TBS-T overnight at 4°C. Membranes were washed in TBS-T, incubated in secondary antibody for ~1 hour, and then washed in TBS-T before incubation in Clarity Western ECL Substrate (Bio-Rad) and detection using the ChemiDoc (Bio-Rad). Primary antibodies used were rabbit anti-PINK1 D8G3 (1:1000; Cell Signaling Technology, 6946, RRID:AB_11179069), rabbit anti–phospho-ubiquitin (Ser^65^) E2J6T (1:1000; Cell Signaling Technology, 62802, RRID:AB_2799632), rabbit anti-Tom20 FL-145 (1:1000; Santa Cruz Biotechnology, sc-11415, RRID:AB_2207533), and mouse anti-Parkin Prk8 (1:1000; Cell Signaling Technology, 4211, RRID:AB_2159920). Secondary antibodies used are goat anti-rabbit horseradish peroxidase (HRP)–conjugated (1:5000; SouthernBiotech, 4010-05, RRID:AB_2632593) and goat anti-mouse HRP-conjugated (1:5000, SouthernBiotech, 1030-05, RRID:AB_2619742). For loading controls, membranes were incubated in hFAB rhodamine anti–glyceraldehyde-3-phosphate dehydrogenase (1:5000; Bio-Rad, 12004167, RRID:AB_2941791) overnight at 4°C, washed in TBS-T and then detected using the ChemiDoc (Bio-Rad).

### Fractionation and ubiquitin phosphorylation assay

Ubiquitin phosphorylation assays using heavy membrane–associated *Hs*PINK1 from OA-treated HeLa *PINK1*^−/−^ cells transfected with *Hs*PINK1 WT, M318A, and M318G were performed as described previously (*12*). HeLa *PINK1*^−/−^ cells (1 × 10^6^) were seeded in 10-cm dishes. After 48 hours, cells were transfected with 5 μg of pcDNA5^d3^-*Hs*PINK1 WT, M318A, or M318G using the Lipofectamine 3000 Transfection Reagent (Invitrogen). Twenty-four hours after transfection, *Hs*PINK1 was stabilized by OA treatment for 2 hours. Cells were harvested by scraping in cold phosphate-buffered saline (PBS) and pelleted by centrifugation at 200*g* for 5 min at 4°C. Cells were permeabilized by incubating for 20 min at 4°C in 1 ml of fractionation buffer [20 mM Hepes (pH 7.4), 250 mM sucrose, 50 mM KCl, and 2.5 mM MgCl_2_] supplemented with 0.025% (w/v) digitonin, 1× cOmplete Protease Inhibitor Cocktail (Roche), and 1× PhosSTOP (Roche). Heavy membrane fractions were pelleted by centrifugation at 14,000*g* for 5 min at 4°C, washed once with 1 ml of fractionation buffer and then resuspended in 100 μl of fractionation buffer. Ubiquitin (15 μM) was added, and membranes were divided into 50 μl of aliquots. The reaction was initiated with 1 mM ATP (Sigma-Aldrich), KTP (Biolog), or an equivalent volume of water and incubated at 30°C for 60 min or the indicated times with gentle agitation. Heavy membranes were pelleted by centrifugation at 14,000*g* for 5 min at 4°C, and samples for the ubiquitin containing supernatant and the *Hs*PINK1 containing heavy membrane pellet were prepared in SDS sample buffer. Western blotting was performed as described above.

### Immunofluorescence assay

HeLa *PINK1*^−/−^ cells stably expressing YFP-Parkin and *Hs*PINK1 were seeded on HistoGrip-coated coverslips 48 hours before treatment with OA and/or 200 μM kinetin for the indicated times. Cells were then fixed with 4% (w/v) paraformaldehyde in 0.1 M phosphate buffer on a rocker for 10 min, rinsed three times with PBS, and permeabilized with 0.1% (v/v) Triton X-100 in PBS for 10 min. After that, samples were blocked with 3% (v/v) goat serum in 0.1% (v/v) Triton X-100/PBS for 15 min and incubated with anti–green fluorescent protein (Thermo Fisher Scientific, A10262, RRID:AB_2534023) and anti-mitochondrial HSP60 (Abcam, ab128567, RRID:AB_11145464) antibodies for 90 min. Following three washes with PBS and subsequent 1 hour of incubation with Alexa Fluor 488 goat anti-chicken IgG (Thermo Fisher Scientific, A32931, RRID:AB_2762843) and Alexa Fluor 647 goat anti-mouse IgG (Thermo Fisher Scientific, A21235, RRID:AB_2535804), the coverslips were washed three times with PBS and mounted with a tris-buffered DABCO-glycerol mounting medium onto glass slides. Imaging of the coverslips was done with an inverted Leica SP8 confocal laser scanning microscope under 63×/1.40 numerical aperture objective (Oil immersion, HC PLAPO, CS2; Leica microsystems). Details on the staining procedure are available at (*49*).

## Supplementary Materials

### This PDF file includes:

Figs. S1 to S9 References
