# Supplementary material for: Interaction of PINK1 with nucleotides and kinetin
Source: Sci Adv. 2024 Jan 19;10(3):eadj7408. doi: 10.1126/sciadv.adj7408 (PMC10798554; doi:10.1126/sciadv.adj7408)
Supplement: Supplementary file 1 — Figs. S1 to S9 References [file sciadv.adj7408_sm.pdf]

Science Advances
AAAS

Supplementary Materials for

Interaction of PINK1 with nucleotides and kinetin

Zhong Yan Gan *et al.*

Corresponding author: David Komander, dk@wehi.edu.au

Sci. Adv. **10**, eadj7408 (2024)
DOI: 10.1126/sciadv.adj7408

This PDF file includes:

Figs. S1 to S9
References

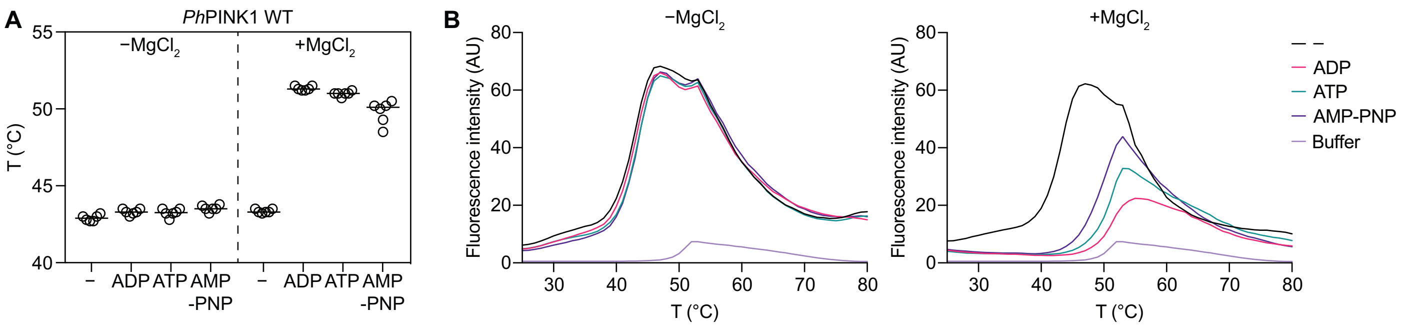
A
*Ph*PINK1 WT
−MgCl$_2$
+MgCl$_2$
T (°C)
55
50
45
40
–
ADP
ATP
AMP-PNP
B
−MgCl$_2$
+MgCl$_2$
Fluorescence intensity (AU)
80
60
40
20
0
30
40
50
60
70
80
T (°C)
–
ADP
ATP
AMP-PNP
Buffer

Supplementary Figure 1. Thermal stability of WT *Ph*PINK1 in the presence of nucleotides and Mg^{2+}.

- **(A)** Melting temperatures of WT *Ph*PINK1 (residues 115–575, monomeric, autophosphorylated) in the presence of ADP, ATP or AMP-PNP, with/without $MgCl_2$. All nucleotides bind and stabilize *Ph*PINK1 in the presence of $MgCl_2$. Experiment was performed three times in technical duplicates.
- **(B)** Representative thermal melt curves for the data in **A**. Note that buffer alone (no protein, nucleotide or $MgCl_2$) produces a small melt curve that overlaps with but is unlikely to notably impact the melt curves of nucleotide-bound *Ph*PINK1. The melt curves for *Ph*PINK1 in the absence of $MgCl_2$ (*left*) and in the presence of $MgCl_2$ (*right*) were generated in the same experiment and are separated for clarity; the buffer curve shown is the same between the two graphs.

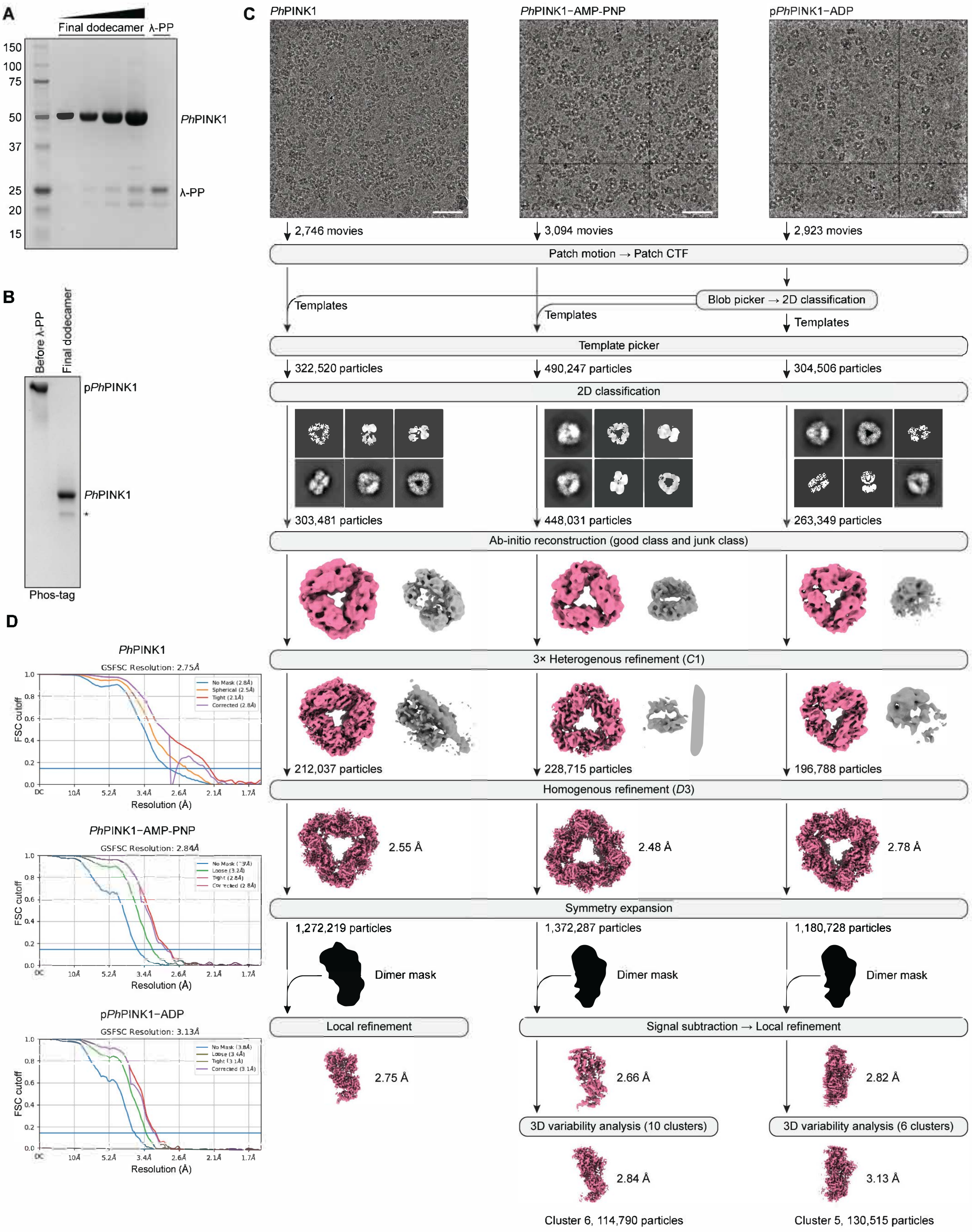
A
Final dodecamer
λ-PP
150
100
75
50
37
25
20
15
PhPINK1
λ-PP
B
Before λ-PP
Final dodecamer
pPhPINK1
PhPINK1
*
Phos-tag
C
PhPINK1
PhPINK1–AMP-PNP
pPhPINK1–ADP
2,746 movies
3,094 movies
2,923 movies
Patch motion → Patch CTF
Blob picker → 2D classification
Templates
Templates
Templates
Template picker
322,520 particles
490,247 particles
304,506 particles
2D classification
303,481 particles
448,031 particles
263,349 particles
Ab-initio reconstruction (good class and junk class)
3× Heterogenous refinement (C1)
212,037 particles
228,715 particles
196,788 particles
Homogenous refinement (D3)
2.55 Å
2.48 Å
2.78 Å
Symmetry expansion
1,272,219 particles
1,372,287 particles
1,180,728 particles
Dimer mask
Dimer mask
Dimer mask
Local refinement
Signal subtraction → Local refinement
2.75 Å
2.66 Å
2.82 Å
3D variability analysis (10 clusters)
3D variability analysis (6 clusters)
2.84 Å
3.13 Å
Cluster 6, 114,790 particles
Cluster 5, 130,515 particles
D
PhPINK1
GSFSC Resolution: 2.75Å
FSC cutoff
Resolution (Å)
PhPINK1–AMP-PNP
GSFSC Resolution: 2.84Å
FSC cutoff
Resolution (Å)
pPhPINK1–ADP
GSFSC Resolution: 3.13Å
FSC cutoff
Resolution (Å)

Supplementary Figure 2. Cryo-EM analysis of nucleotide-bound *Ph*PINK1.

- **(A)** The final purified dephosphorylated WT *Ph*PINK1 dodecamer run at increasing concentrations on SDS-PAGE, revealing a minor contaminating species at approximately 25 kDa, likely to be λ-PP.
- **(B)** Phos-tag analysis (7.5% gel) of *Ph*PINK1 before dephosphorylation by λ-PP, and after purification of the final *Ph*PINK1 dodecamer. Note, the faster migrating band (labelled with an asterisk) is likely to be fully dephosphorylated *Ph*PINK1, while the major band is presumed to be *Ph*PINK1 pThr305 given that density for pThr305 can be seen in subsequent cryo-EM reconstructions (Supplementary Figure 3C). Dephosphorylation of pThr305 is likely prevented by the adjacent Pro306 (*20*, *50*). Phosphorylation of Thr305 is a by-product of non-specific *Ph*PINK1 autophosphorylation during expression in bacteria(*20*).
- **(C)** Cryo-EM processing pipeline for the nucleotide-free, AMP-PNP-bound and ADP-bound *Ph*PINK1 dimers. Representative micrographs and select 2D classes are shown. Scalebar, 50 nm.
- **(D)** Gold standard Fourier shell correlation (GSFSC) curves for each of the final maps in **C**.

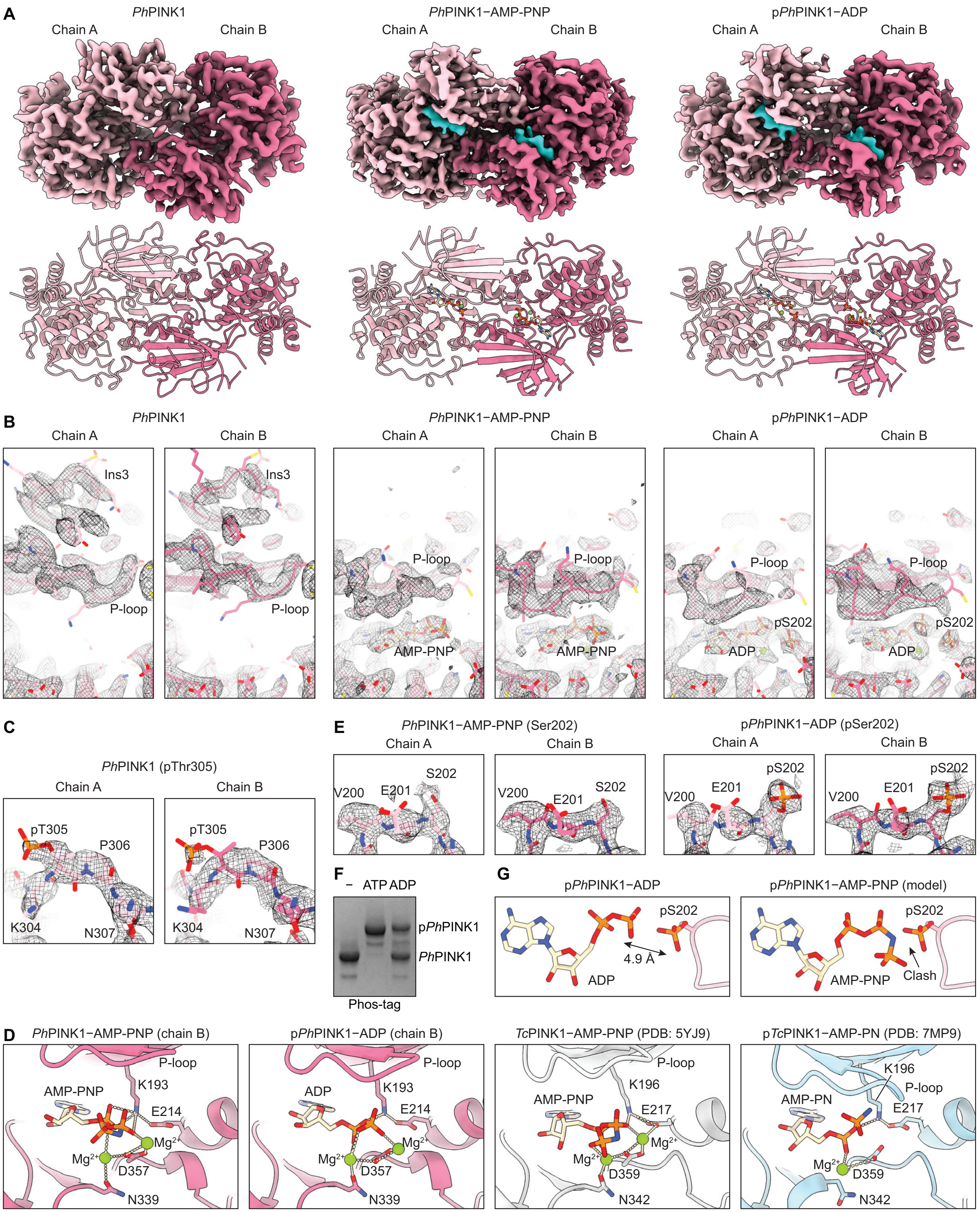
A
PhPINK1
PhPINK1–AMP-PNP
pPhPINK1–ADP
Chain A
Chain B
B
Ins3
P-loop
AMP-PNP
ADP
pS202
C
PhPINK1 (pThr305)
pT305
P306
K304
N307
E
PhPINK1–AMP-PNP (Ser202)
pPhPINK1–ADP (pSer202)
V200
E201
S202
F
–
ATP
ADP
pPhPINK1
PhPINK1
Phos-tag
G
pPhPINK1–ADP
pPhPINK1–AMP-PNP (model)
4.9 Å
Clash
D
PhPINK1–AMP-PNP (chain B)
pPhPINK1–ADP (chain B)
TcPINK1–AMP-PNP (PDB: 5YJ9)
pTcPINK1–AMP-PN (PDB: 7MP9)
K193
E214
Mg2+
D357
N339
K196
E217
D359
N342
AMP-PN

Supplementary Figure 3. Cryo-EM structure of the nucleotide-bound *Ph*PINK1 dimers.

- **(A)** Cryo-EM densities (*top*) and cartoon model (*bottom*) of the nucleotide-free, AMP-PNP-bound and ADP-bound *Ph*PINK1 dimers. Each *Ph*PINK1 monomer is coloured in a different shade of pink, and density for the nucleotide and Mg^{2+} ions is coloured in cyan. Densities for nucleotide-free, AMP-PNP-bound and ADP-bound *Ph*PINK1 are contoured to levels 0.35, 0.12 and 0.12, respectively.
- **(B)** Zoomed view of the ATP binding site, P-loop and insertion-3 of chains A and B of each *Ph*PINK1 dimer. Residue side chains and nucleotides are shown as sticks, and Mg^{2+} as green spheres. Corresponding densities are displayed as a mesh.
- **(C)** Density for phosphorylated Thr305 can be seen in each chain of the nucleotide-free *Ph*PINK1 dimer, consistent with Phos-tag analysis of the purified dephosphorylated *Ph*PINK1 dodecamer that was used for cryo-EM (see Supplementary Figure 2B and legend) and previous observations (*20*).
- **(D)** Comparison between the binding of *Ph*PINK1 to AMP-PNP and ADP (only chain B is shown) and the binding of *Tc*PINK1 to AMP-PNP (PDB: 5YJ9, (*22*)) and AMP-PN (PDB: 7MP9, (*13*)). Polar interactions are shown as dotted lines. *Ph*PINK1 and *Tc*PINK1 bind nucleotides via similar interactions.
- **(E)** Density for phosphorylated Ser202 can be seen for both chains of p*Ph*PINK1–ADP, but not *Ph*PINK1–AMP-PNP.
- **(F)** The dephosphorylated *Ph*PINK1 dodecamer was incubated with 10 mM ADP (or ATP as a positive control) and 10 mM $MgCl_2$ for 10 min on ice. The ADP stock used in the experiment was the same stock used for cryo-EM analysis. Phos-tag analysis (7.5% gel) revealed that ADP incubation leads to *Ph*PINK1 autophosphorylation, indicating that the ADP stock is contaminated with ATP and explaining the density for pSer202 seen in cryo-EM reconstructions (see **E**).
- **(G)** *Left*, the position of ADP (chain B) relative to pSer202 (chain A) in the p*Ph*PINK1–ADP structure. *Right*, AMP-PNP (chain B) from the *Ph*PINK1–AMP-PNP structure is modelled relative to pSer202 (chain A) from the p*Ph*PINK1–ADP structure.

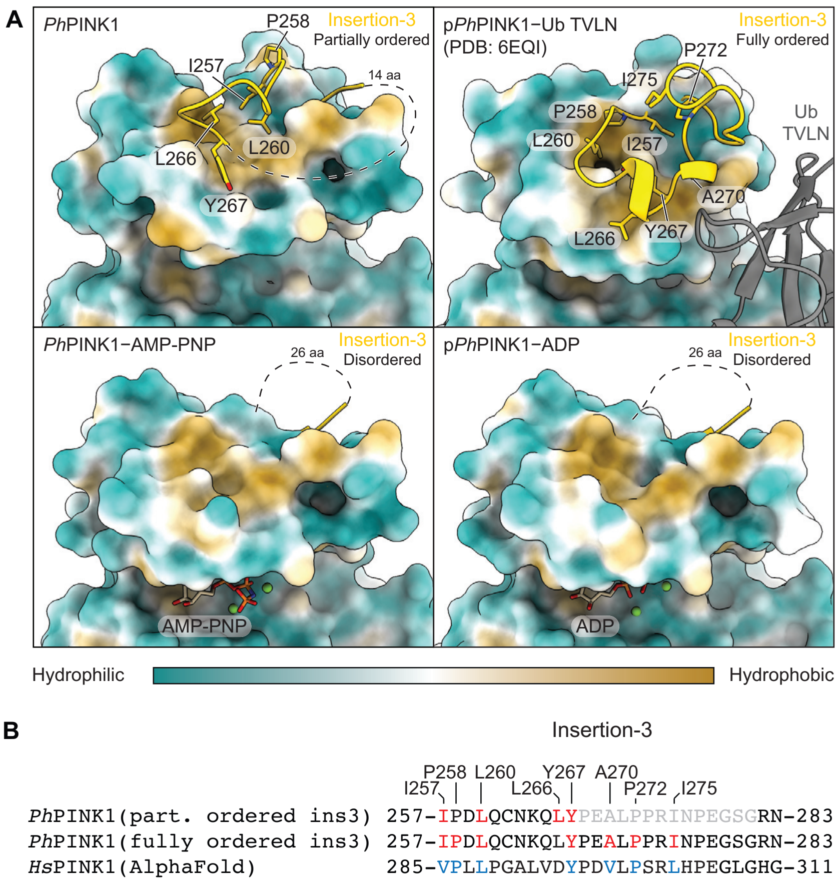

A
PhPINK1
Insertion-3
Partially ordered
P258
I257
14 aa
L260
L266
Y267
pPhPINK1–Ub TVLN
(PDB: 6EQI)
Insertion-3
Fully ordered
P272
I275
P258
L260
I257
Ub
TVLN
A270
L266
Y267
PhPINK1–AMP-PNP
26 aa
Insertion-3
Disordered
AMP-PNP
pPhPINK1–ADP
26 aa
Insertion-3
Disordered
ADP
Hydrophilic
Hydrophobic
B
Insertion-3
P258 L260 Y267 A270
I257 L266 P272 I275
PhPINK1(part. ordered ins3) 257-IPDLQCNKQLYPEALPPRINPEGSGRN-283
PhPINK1(fully ordered ins3) 257-IPDLQCNKQLYPEALPPRINPEGSGRN-283
HsPINK1(AlphaFold) 285-VPLLPGALVDYPDVLPSRLHPEGLGHG-311

Supplementary Figure 4. The hydrophobic interface between insertion-3 and the N-lobe.

(A) Hydrophobicity of the N-lobe of *Ph*PINK1 in the nucleotide-free, AMP-PNP-bound and ADP-bound states (only chain B is shown), and the phosphorylated and ubiquitin-bound *Ph*PINK1 complex (PDB: 6EQI, (*20*)). Insertion-3, shown in cartoon representation, shields a hydrophobic patch in the N-lobe. Hydrophobic residues are shown as sticks. aa, amino acids.

(B) Sequence alignment of insertion-3 from *Ph*PINK1 and *Hs*PINK1. *Ph*PINK1 residues involved in the hydrophobic insertion-3–N-lobe interaction are highlighted in red. *Hs*PINK1 residues predicted by AlphaFold (*51*, *52*) to be involved in the hydrophobic insertion-3–N-lobe interaction are highlighted in blue. Residues in light grey are disordered in the structure. ins3, insertion-3.

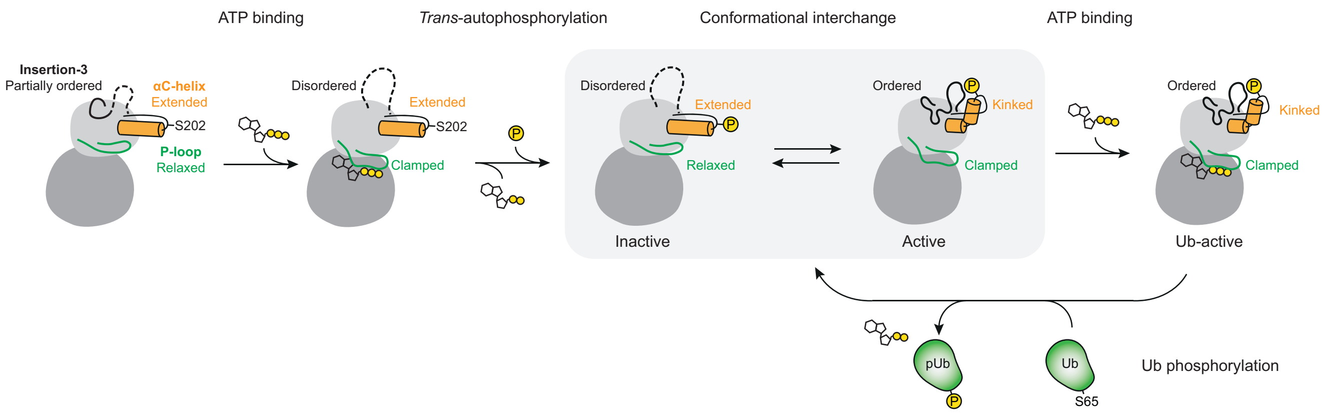

ATP binding
Trans-autophosphorylation
Conformational interchange
ATP binding
Insertion-3
Partially ordered
αC-helix
Extended
S202
P-loop
Relaxed
Disordered
Extended
S202
Clamped
P
Disordered
Extended
P
Relaxed
Inactive
Ordered
P
Kinked
Clamped
Active
Ordered
P
Kinked
Clamped
Ub-active
pUb
P
Ub
S65
Ub phosphorylation

Supplementary Figure 5. Model of how ATP binding contributes to PINK1 activation and ubiquitin phosphorylation.

Unphosphorylated PINK1 dimerizes and utilizes ATP to *trans*-autophosphorylate at Ser202. The resulting phosphorylated PINK1 can adopt two conformations, an inactive conformation that cannot bind ubiquitin (relaxed P-loop, extended αC-helix, disordered insertion-3) and an active conformation that can bind ubiquitin (clamped P-loop, kinked αC-helix, ordered insertion-3). Binding of ATP to phosphorylated PINK1 stabilizes the active conformation via its interaction with the clamped P-loop, enabling efficient ubiquitin recognition and phosphorylation by PINK1.

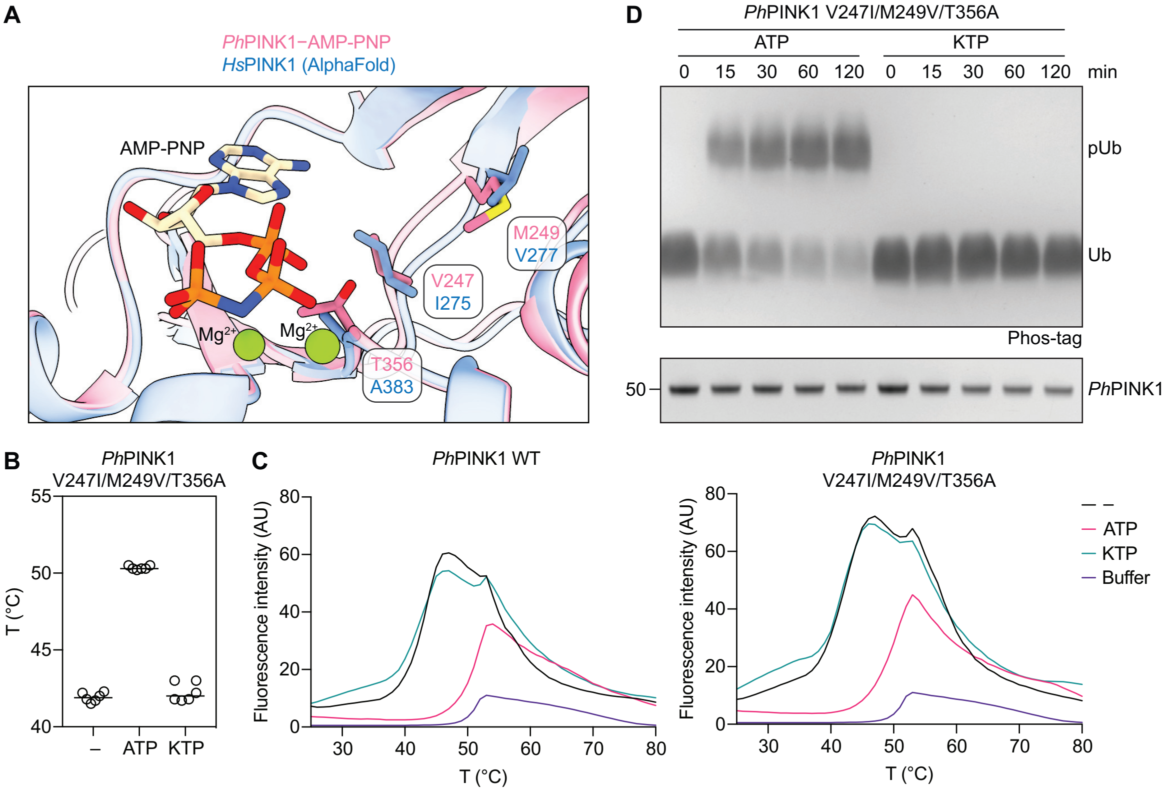

A
PhPINK1–AMP-PNP
HsPINK1 (AlphaFold)
AMP-PNP
Mg2+
Mg2+
M249
V277
V247
I275
T356
A383
D
PhPINK1 V247I/M249V/T356A
ATP
KTP
0 15 30 60 120 0 15 30 60 120 min
pUb
Ub
Phos-tag
50
PhPINK1
B
PhPINK1
V247I/M249V/T356A
55
50
45
40
T (°C)
– ATP KTP
C
PhPINK1 WT
Fluorescence intensity (AU)
80
60
40
20
0
30 40 50 60 70 80
T (°C)
PhPINK1
V247I/M249V/T356A
–
ATP
KTP
Buffer

Supplementary Figure 6. A humanized mutant of *Ph*PINK1 does not bind KTP.

- **(A)** Superimposed ATP binding sites of *Ph*PINK1–AMP-PNP and *Hs*PINK1 (AlphaFold; (*51*, *52*)). The N-lobe β1-, β2- and β3-strands are hidden for clarity. Three AMP-PNP-proximal residues in *Ph*PINK1 (Val247, Met249, Thr356) were mutated into their equivalent *Hs*PINK1 counterparts to generate a humanized version of *Ph*PINK1 for the experiments in **B–D**.
- **(B)** Melting temperatures of *Ph*PINK1 V247I/M249V/T356A in the presence of ATP or KTP. No increase in melting temperature is observed for KTP, indicating an absence of interaction. Experiment was performed three times in duplicate.
- **(C)** Representative thermal melt curves for the data in **B** and Figure 3B. The buffer curve was generated in the absence of protein and nucleotide. The melt curves for WT *Ph*PINK1 and *Ph*PINK1 V247I/M249V/T356A were separated into different graphs for clarity, and the buffer curve shown is the same between the two graphs.
- **(D)** Ubiquitin phosphorylation assay using *Ph*PINK1 V247I/M249V/T356A in the presence of ATP or KTP, analyzed on a Phos-tag gel. *Ph*PINK1 activity was not detected when KTP was supplied. Experiment was performed in triplicate.

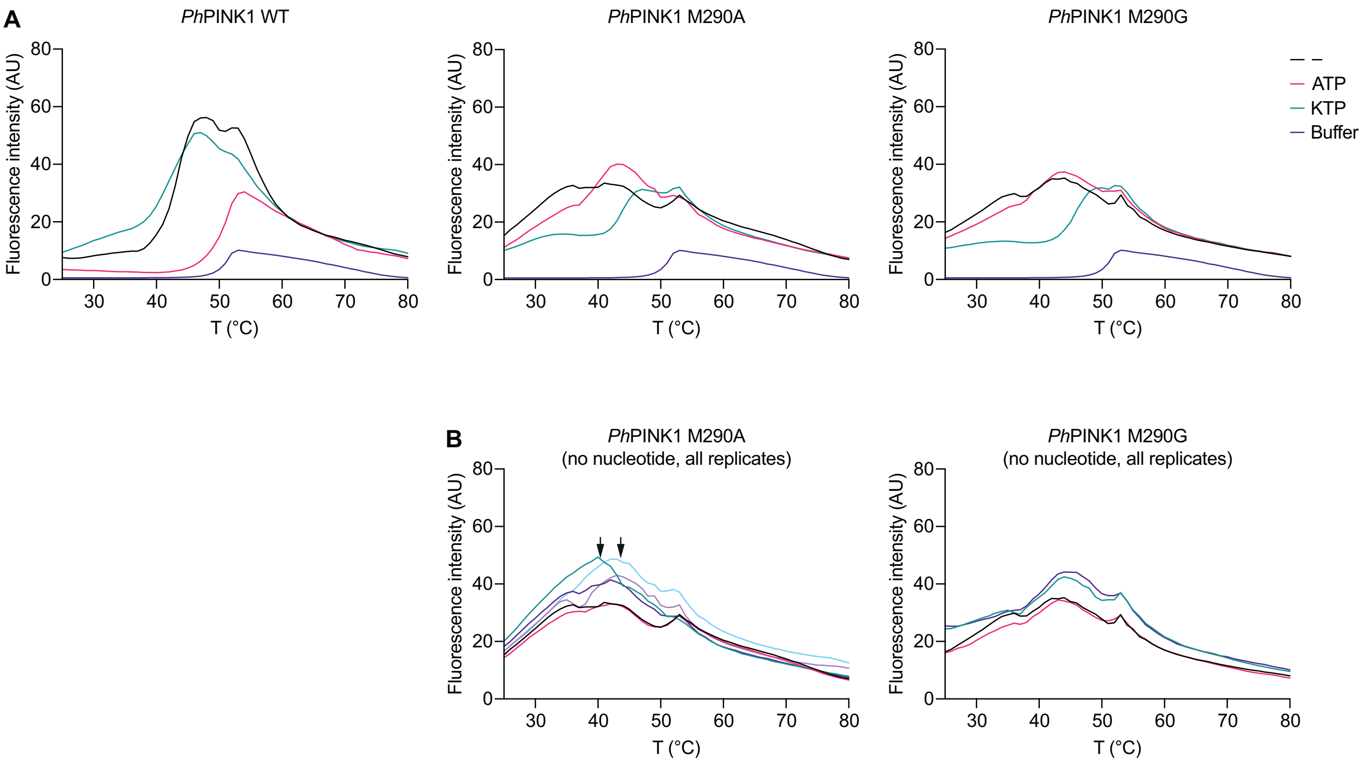
A
PhPINK1 WT
PhPINK1 M290A
PhPINK1 M290G
Fluorescence intensity (AU)
T (°C)
–
ATP
KTP
Buffer
B
PhPINK1 M290A
(no nucleotide, all replicates)
PhPINK1 M290G
(no nucleotide, all replicates)

Supplementary Figure 7. Instability of *Ph*PINK1 M290A and M290G.

(A) Representative thermal melt curves for experiment in Figure 3G. *Ph*PINK1 M290A and M290G (*middle* and *right*, respectively) in the absence of nucleotide (blue curves) or in the presence of ATP (red curves) show relatively indistinct melt curves, indicating low stability of either mutant. Addition of KTP resulted in better defined melt curves (green curves), consistent with nucleotide binding increasing the stability of the M290A and M290G mutants. The experiment was repeated a total of two (WT, M290G) or three (M290A) times in technical duplicates. Melting temperatures could only be calculated for a subset of replicates (see **B**). Melt curves for all three *Ph*PINK1 variants were separated for clarity; the buffer curve shown is the same between the three graphs.

(B) All replicate melt curves for M290A and M290G in the absence of nucleotide are shown. While melting temperatures could not be determined for two of the M290A curves (arrows), the other four curves displayed a small inflection at ~40 °C that was sufficient for melting temperature calculation.

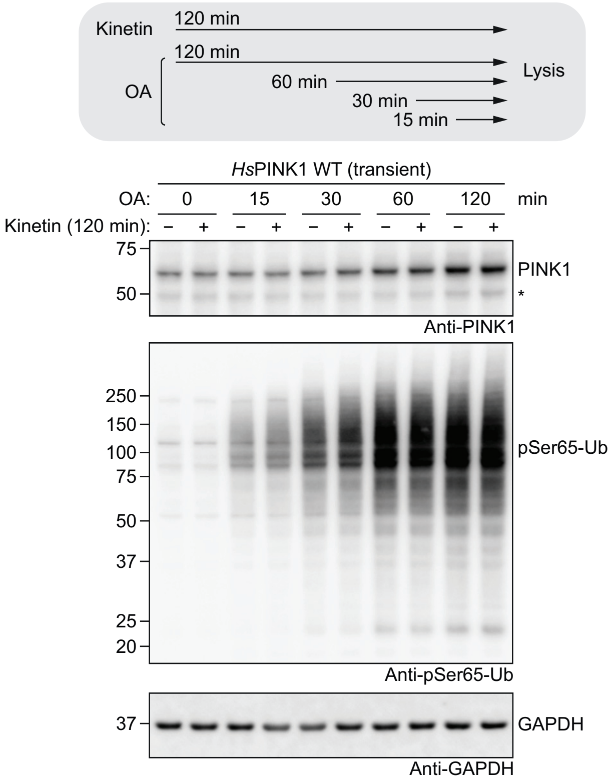
Kinetin
120 min
120 min
OA
60 min
30 min
15 min
Lysis
HsPINK1 WT (transient)
OA:
0
15
30
60
120
min
Kinetin (120 min):
75
50
PINK1
*
Anti-PINK1
250
150
100
75
50
37
25
20
pSer65-Ub
Anti-pSer65-Ub
37
GAPDH
Anti-GAPDH

Supplementary Figure 8. Kinetin does not appear to affect the ubiquitin kinase activity of *Hs*PINK1 WT.

HeLa *PINK1*$^{-/-}$ YFP–Parkin cells transiently expressing WT *Hs*PINK1 were co-treated with OA and 200 µM kinetin according to the schematic (top panel). Immunoblotting shows that kinetin has no effect on WT *Hs*PINK1-mediated ubiquitin phosphorylation. The asterisk indicates the 52-kDa PARL-cleaved PINK1. Experiment was performed in triplicate.

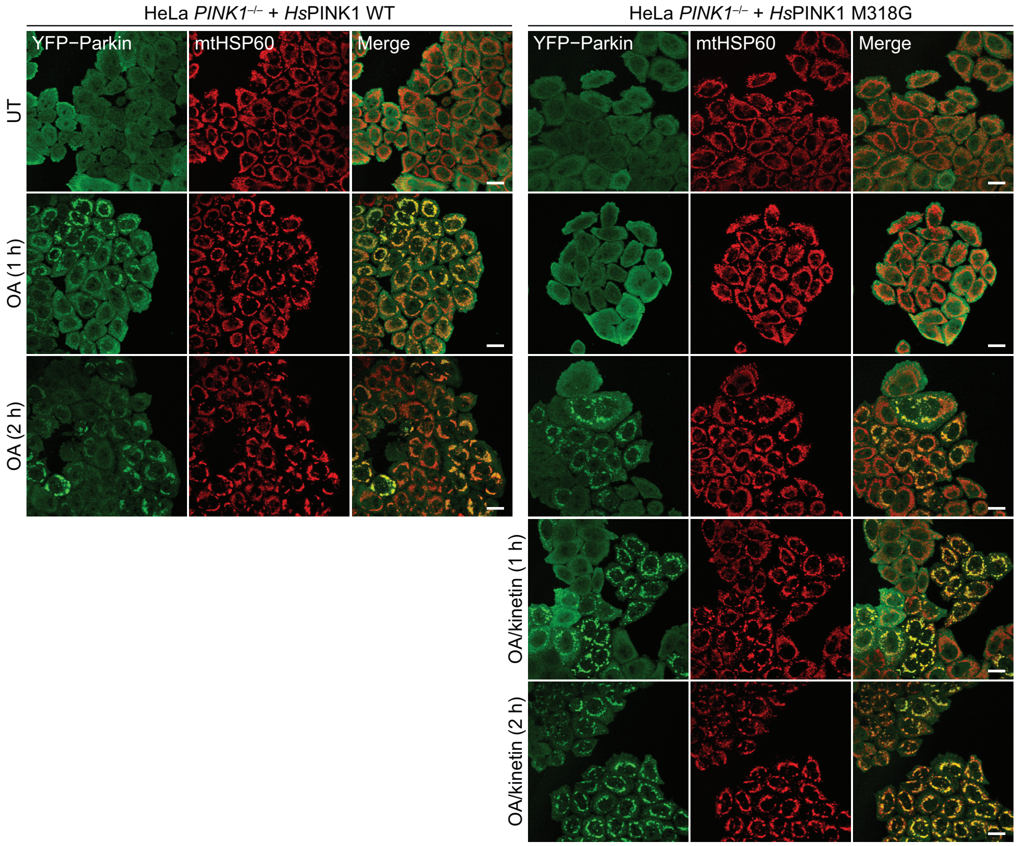

HeLa PINK1−/− + HsPINK1 WT
HeLa PINK1−/− + HsPINK1 M318G
YFP−Parkin
mtHSP60
Merge
YFP−Parkin
mtHSP60
Merge
UT
OA (1 h)
OA (2 h)
OA/kinetin (1 h)
OA/kinetin (2 h)

Supplementary Figure 9. Further immunofluorescence analysis of Parkin translocation.

Repeat of Figure 5 but showing the 2 h OA/kinetin treatment timepoints. At 2 h, OA treatment alone induced YFP–Parkin translocation in *Hs*PINK1 M318G-expressing cells. In this repeat, cells were additionally immunostained for PINK1, but fluorescence signal consistent with PINK1 was not detected and therefore not shown. Scalebar, 20 μm. Experiment was performed in duplicate with Figure 5.

REFERENCES AND NOTES

1. M. F. Schmidt, Z. Y. Gan, D. Komander, G. Dewson, Ubiquitin signalling in neurodegeneration: Mechanisms and therapeutic opportunities. *Cell Death Differ.* **28**, 570–590 (2021).

2. A. Legati, D. Ghezzi, Parkinson's disease, Parkinsonisms, and mitochondria: The role of nuclear and mitochondrial DNA. *Curr. Neurol. Neurosci. Rep.* **23**, 131–147 (2023).

3. O. Corti, S. Lesage, A. Brice, What genetics tells us about the causes and mechanisms of Parkinson's disease. *Physiol. Rev.* **91**, 1161–1218 (2011).

4. T. Kitada, S. Asakawa, N. Hattori, H. Matsumine, Y. Yamamura, S. Minoshima, M. Yokochi, Y. Mizuno, N. Shimizu, Mutations in the parkin gene cause autosomal recessive juvenile parkinsonism. *Nature* **392**, 605–608 (1998).

5. E. M. Valente, P. M. Abou-Sleiman, V. Caputo, M. M. K. Muqit, K. Harvey, S. Gispert, Z. Ali, D. D. Turco, A. R. Bentivoglio, D. G. Healy, A. Albanese, R. Nussbaum, R. González-Maldonado, T. Deller, S. Salvi, P. Cortelli, W. P. Gilks, D. S. Latchman, R. J. Harvey, B. Dallapiccola, G. Auburger, N. W. Wood, Hereditary early-onset Parkinson's disease caused by mutations in PINK1. *Science (New York, NY)* **304**, 1158–1160 (2004).

6. J. W. Harper, A. Ordureau, J.-M. Heo, Building and decoding ubiquitin chains for mitophagy. *Nat. Rev. Mol. Cell Biol.* **19**, 93–108 (2018).

7. T. N. Nguyen, B. S. Padman, M. Lazarou, Deciphering the molecular signals of PINK1/Parkin mitophagy. *Trends Cell Biol.* **26**, 733–744 (2016).

8. S. Sekine, R. J. Youle, PINK1 import regulation; a fine system to convey mitochondrial stress to the cytosol. *BMC Biol.* **16**, 2 (2018).

9. M. Lazarou, S. M. Jin, L. A. Kane, R. J. Youle, Role of PINK1 binding to the TOM complex and alternate intracellular membranes in recruitment and activation of the E3 ligase Parkin. *Dev. Cell* **22**, 320–333 (2012).

10. K. Okatsu, T. Oka, M. Iguchi, K. Imamura, H. Kosako, N. Tani, M. Kimura, E. Go, F. Koyano, M. Funayama, K. Shiba-Fukushima, S. Sato, H. Shimizu, Y. Fukunaga, H. Taniguchi, M. Komatsu, N. Hattori, K. Mihara, K. Tanaka, N. Matsuda, PINK1 autophosphorylation upon membrane potential dissipation is essential for Parkin recruitment to damaged mitochondria. *Nat. Commun.* **3**, 1016 (2012).

11. K. Okatsu, M. Uno, F. Koyano, E. Go, M. Kimura, T. Oka, K. Tanaka, N. Matsuda, A dimeric PINK1-containing complex on depolarized mitochondria stimulates Parkin recruitment. *J. Biol. Chem.* **288**, 36372–36384 (2013).

12. Z. Y. Gan, S. Callegari, S. A. Cobbold, T. R. Cotton, M. J. Mlodzianoski, A. F. Schubert, N. D. Geoghegan, K. L. Rogers, A. Leis, G. Dewson, A. Glukhova, D. Komander, Activation mechanism of PINK1. *Nature* **602**, 328–335 (2022).

13. S. Rasool, S. Veyron, N. Soya, M. A. Eldeeb, G. L. Lukacs, E. A. Fon, J.-F. Trempe, Mechanism of PINK1 activation by autophosphorylation and insights into assembly on the TOM complex. *Mol. Cell* **82**, 44–59.e6 (2022).

14. F. Koyano, K. Okatsu, H. Kosako, Y. Tamura, E. Go, M. Kimura, Y. Kimura, H. Tsuchiya, H. Yoshihara, T. Hirokawa, T. Endo, E. A. Fon, J.-F. Trempe, Y. Saeki, K. Tanaka, N. Matsuda, Ubiquitin is phosphorylated by PINK1 to activate parkin. *Nature* **510**, 162–166 (2014).

15. A. Kazlauskaite, C. Kondapalli, R. Gourlay, D. G. Campbell, M. S. Ritorto, K. Hofmann, D. R. Alessi, A. Knebel, M. Trost, M. M. K. Muqit, Parkin is activated by PINK1-dependent phosphorylation of ubiquitin at Ser^{65}. *Biochem. J.* **460**, 127–141 (2014).

16. L. A. Kane, M. Lazarou, A. I. Fogel, Y. Li, K. Yamano, S. A. Sarraf, S. Banerjee, R. J. Youle, PINK1 phosphorylates ubiquitin to activate Parkin E3 ubiquitin ligase activity. *J. Cell Biol.* **205**, 143–153 (2014).

17. A. Ordureau, S. A. Sarraf, D. M. Duda, J.-M. Heo, M. P. Jedrychowski, V. O. Sviderskiy, J. L. Olszewski, J. T. Koerber, T. Xie, S. A. Beausoleil, J. A. Wells, S. P. Gygi, B. A. Schulman,

J. W. Harper, Quantitative proteomics reveal a feedforward mechanism for mitochondrial PARKIN translocation and ubiquitin chain synthesis. *Mol. Cell* **56**, 360–375 (2014).

18. T. Wauer, K. N. Swatek, J. L. Wagstaff, C. Gladkova, J. N. Pruneda, M. A. Michel, M. Gersch, C. M. Johnson, S. M. Freund, D. Komander, Ubiquitin Ser65 phosphorylation affects ubiquitin structure, chain assembly and hydrolysis. *EMBO J.* **34**, 307–325 (2015).

19. C. Gladkova, S. L. Maslen, J. M. Skehel, D. Komander, Mechanism of parkin activation by PINK1. *Nature* **559**, 410–414 (2018).

20. A. F. Schubert, C. Gladkova, E. Pardon, J. L. Wagstaff, S. M. V. Freund, J. Steyaert, S. L. Maslen, D. Komander, Structure of PINK1 in complex with its substrate ubiquitin. *Nature* **552**, 51–56 (2017).

21. A. Kumar, J. Tamjar, A. D. Waddell, H. I. Woodroof, O. G. Raimi, A. M. Shaw, M. Peggie, M. M. Muqit, D. M. van Aalten, Structure of PINK1 and mechanisms of Parkinson's disease-associated mutations. *eLife* **6**, e29985 (2017).

22. K. Okatsu, Y. Sato, K. Yamano, N. Matsuda, L. Negishi, A. Takahashi, A. Yamagata, S. Goto-Ito, M. Mishima, Y. Ito, T. Oka, K. Tanaka, S. Fukai, Structural insights into ubiquitin phosphorylation by PINK1. *Sci Rep-uk* **8**, 10382 (2018).

23. H. I. Woodroof, J. H. Pogson, M. Begley, L. C. Cantley, M. Deak, D. G. Campbell, D. M. F. van Aalten, A. J. Whitworth, D. R. Alessi, M. M. K. Muqit, Discovery of catalytically active orthologues of the Parkinson's disease kinase PINK1: Analysis of substrate specificity and impact of mutations. *Open Biol.* **1**, 110012 (2011).

24. S. Padmanabhan, N. K. Polinski, L. B. Menalled, M. A. S. Baptista, B. K. Fiske, The Michael J. Fox Foundation for Parkinson's research strategy to advance therapeutic development of PINK1 and Parkin. *Biomol* **9**, 296 (2019).

25. S. Miller, M. M. K. Muqit, Therapeutic approaches to enhance PINK1/Parkin mediated mitophagy for the treatment of Parkinson's disease. *Neurosci. Lett.* **705**, 7–13 (2019).

26. N. T. Hertz, A. Berthet, M. L. Sos, K. S. Thorn, A. L. Burlingame, K. Nakamura, K. M. Shokat, A neo-substrate that amplifies catalytic activity of parkinson's-disease-related kinase PINK1. *Cell* **154**, 737–747 (2013).

27. A. Ordureau, J. A. Paulo, W. Zhang, T. Ahfeldt, J. Zhang, E. F. Cohn, Z. Hou, J.-M. Heo, L. L. Rubin, S. S. Sidhu, S. P. Gygi, J. W. Harper, Dynamics of PARKIN-dependent mitochondrial ubiquitylation in induced neurons and model systems revealed by digital snapshot proteomics. *Mol. Cell* **70**, 211–227.e8 (2018).

28. R. M. Chin, R. Rakhit, D. Ditsworth, C. Wang, J. Bartholomeus, S. Liu, A. Mody, A. Laishu, A. Eastes, C. Tai, R. Y. Kim, J. Li, S. Hansberry, S. Khasnavis, V. Rafalski, D. Herendeen, V. Garda, J. Phung, D. de Roulet, A. Ordureau, J. W. Harper, S. Johnstone, J. Stöhr, N. T. Hertz, Pharmacological PINK1 activation ameliorates pathology in Parkinson's disease models. bioRxiv 2023.02.14.528378 (2023).

29. A. Punjani, J. L. Rubinstein, D. J. Fleet, M. A. Brubaker, cryoSPARC: Algorithms for rapid unsupervised cryo-EM structure determination. *Nat. Methods* **14**, 290–296 (2017).

30. S. S. Taylor, A. P. Kornev, Protein kinases: Evolution of dynamic regulatory proteins. *Trends Biochem. Sci.* **36**, 65–77 (2011).

31. K. Islam, The bump-and-hole tactic: Expanding the scope of chemical genetics. *Cell Chem. Biol.* **25**, 1171–1184 (2018).

32. O. A. Lambourne, S. Bell, L. P. Wilhelm, E. B. Yarbrough, G. G. Holly, O. M. Russell, A. M. Alghamdi, A. M. Fdel, C. Varricchio, E. L. Lane, I. G. Ganley, A. T. Jones, M. S. Goldberg, Y. Mehellou, PINK1-dependent mitophagy inhibits elevated ubiquitin phosphorylation caused by mitochondrial damage. *J. Med. Chem.* **66**, 7645–7656. (2023).

33. L. Osgerby, Y.-C. Lai, P. J. Thornton, J. Amalfitano, C. S. L. Duff, I. Jabeen, H. Kadri, A. Miccoli, J. H. R. Tucker, M. M. K. Muqit, Y. Mehellou, Kinetin riboside and its ProTides activate the Parkinson's disease associated PTEN-induced putative kinase 1 (PINK1) independent of mitochondrial depolarization. *J. Med. Chem.* **60**, 3518–3524 (2017).

34. R. C. C. Hengeveld, N. T. Hertz, M. J. M. Vromans, C. Zhang, A. L. Burlingame, K. M. Shokat, S. M. A. Lens, Development of a chemical genetic approach for human Aurora B kinase identifies novel substrates of the chromosomal passenger complex. *Mol. Cell. Proteomics* **11**, 47–59 (2012).

35. R. S. Levin, N. T. Hertz, A. L. Burlingame, K. M. Shokat, S. Mukherjee, Innate immunity kinase TAK1 phosphorylates Rab1 on a hotspot for posttranslational modifications by host and pathogen. *Proc. Natl. Acad. Sci.* **113**, E4776–E4783 (2016).

36. A. W. Lin, K. K. Gill, M. S. Castañeda, I. Matucci, N. Eder, S. Claxton, H. Flynn, A. P. Snijders, R. George, S. K. Ultanir, Chemical genetic identification of GAK substrates reveals its role in regulating Na+/K+-ATPase. *Life Sci Alliance* **1**, e201800118 (2018).

37. N. L. Maas, N. Singh, J. A. Diehl, Generation and characterization of an analog-sensitive PERK allele. *Cancer Biol. Ther.* **15**, 1106–1111 (2014).

38. S. Sharma, T. Zhang, W. Michowski, V. W. Rebecca, M. Xiao, R. Ferretti, J. M. Suski, R. T. Bronson, J. A. Paulo, D. Frederick, A. Fassl, G. M. Boland, Y. Geng, J. A. Lees, R. H. Medema, M. Herlyn, S. P. Gygi, P. Sicinski, Targeting the cyclin-dependent kinase 5 in metastatic melanoma. *Proc. Natl. Acad. Sci.* **117**, 8001–8012 (2020).

39. A. D. Yildirim, M. Citir, A. E. Dogan, Z. Veli, Z. Yildirim, O. Tufanli, A. Traynor-Kaplan, C. Schultz, E. Erbay, ER stress-induced sphingosine-1-phosphate lyase phosphorylation potentiates the mitochondrial unfolded protein response. *J. Lipid Res.* **63**, 100279 (2022).

40. X. Dong, J. Sun, W. Miao, C. A. Chang, Y. Wang, Proteome-wide Characterizations of N6-methyl-adenosine triphosphate- and N^6-furfuryl-Adenosine triphosphate-binding capabilities of kinases. *Anal. Chem.* **93**, 13251–13259 (2021).

41. D. P. Narendra, C. Wang, R. J. Youle, J. E. Walker, PINK1 rendered temperature sensitive by disease-associated and engineered mutations. *Hum. Mol. Genet.* **22**, 2572–2589 (2013).

42. A. C. Bishop, J. A. Ubersax, D. T. Petsch, D. P. Matheos, N. S. Gray, J. Blethrow, E. Shimizu, J. Z. Tsien, P. G. Schultz, M. D. Rose, J. L. Wood, D. O. Morgan, K. M. Shokat, A

chemical switch for inhibitor-sensitive alleles of any protein kinase. *Nature* **407**, 395–401 (2000).

43. C. Zhang, S. Lee, Y. Peng, E. Bunker, C. Shen, E. Giaime, J. Shen, J. Shen, Z. Zhou, X. Liu, A chemical genetic approach to probe the function of PINK1 in regulating mitochondrial dynamics. *Cell Res.* **25**, 394–397 (2015).

44. N. S. Berrow, D. Alderton, S. Sainsbury, J. Nettleship, R. Assenberg, N. Rahman, D. I. Stuart, R. J. Owens, A versatile ligation-independent cloning method suitable for high-throughput expression screening applications. *Nucleic Acids Res.* **35**, e45 (2007).

45. E. F. Pettersen, T. D. Goddard, C. C. Huang, E. C. Meng, G. S. Couch, T. I. Croll, J. H. Morris, T. E. Ferrin, UCSF ChimeraX: Structure visualization for researchers, educators, and developers. *Protein Sci.* **30**, 70–82 (2021).

46. P. Emsley, B. Lohkamp, W. G. Scott, K. Cowtan, Features and development of Coot. *Acta Crystallogr. D Biol. Crystallogr.* **66**, 486–501 (2010).

47. P. D. Adams, P. V. Afonine, G. Bunkóczi, V. B. Chen, N. Echols, J. J. Headd, L.-W. Hung, S. Jain, G. J. Kapral, R. W. G. Kunstleve, A. J. McCoy, N. W. Moriarty, R. D. Oeffner, R. J. Read, D. C. Richardson, J. S. Richardson, T. C. Terwilliger, P. H. Zwart, The Phenix software for automated determination of macromolecular structures. *Methods (San Diego, Calif)* **55**, 94–106 (2011).

48. M. Lazarou, D. A. Sliter, L. A. Kane, S. A. Sarraf, C. Wang, J. L. Burman, D. P. Sideris, A. I. Fogel, R. J. Youle, The ubiquitin kinase PINK1 recruits autophagy receptors to induce mitophagy. *Nature* **524**, 309–314 (2015).

49. T. N. Nguyen, Immunofluorescence assay (IFA) (2023); https://doi.org/10.17504/protocols.io.5qpvobz99l4o/v1.

50. T. Ansai, L. C. Dupuy, S. Barik, Interactions between a minimal protein serine/threonine phosphatase and its phosphopeptide substrate sequence. *J. Biol. Chem.* **271**, 24401–24407 (1996).

51. J. Jumper, R. Evans, A. Pritzel, T. Green, M. Figurnov, O. Ronneberger, K. Tunyasuvunakool, R. Bates, A. Žídek, A. Potapenko, A. Bridgland, C. Meyer, S. A. A. Kohl, A. J. Ballard, A. Cowie, B. Romera-Paredes, S. Nikolov, R. Jain, J. Adler, T. Back, S. Petersen, D. Reiman, E. Clancy, M. Zielinski, M. Steinegger, M. Pacholska, T. Berghammer, S. Bodenstein, D. Silver, O. Vinyals, A. W. Senior, K. Kavukcuoglu, P. Kohli, D. Hassabis, Highly accurate protein structure prediction with AlphaFold. *Nature* **596**, 583–589 (2021).

52. K. Tunyasuvunakool, J. Adler, Z. Wu, T. Green, M. Zielinski, A. Žídek, A. Bridgland, A. Cowie, C. Meyer, A. Laydon, S. Velankar, G. J. Kleywegt, A. Bateman, R. Evans, A. Pritzel, M. Figurnov, O. Ronneberger, R. Bates, S. A. A. Kohl, A. Potapenko, A. J. Ballard, B. Romera-Paredes, S. Nikolov, R. Jain, E. Clancy, D. Reiman, S. Petersen, A. W. Senior, K. Kavukcuoglu, E. Birney, P. Kohli, J. Jumper, D. Hassabis, Highly accurate protein structure prediction for the human proteome. *Nature* **596**, 590–596 (2021).
